# Human Adipose Stem Cells (hASCs) Grown on Biodegradable Microcarriers in Serum- and Xeno-Free Medium Preserve Their Undifferentiated Status

**DOI:** 10.3390/jfb12020025

**Published:** 2021-04-16

**Authors:** Francesco Muoio, Stefano Panella, Valentin Jossen, Matias Lindner, Yves Harder, Michele Müller, Regine Eibl, Tiziano Tallone

**Affiliations:** 1 Foundation for Cardiological Research and Education (FCRE), Cardiocentro Ticino Foundation, 6807 Taverne, Switzerland; francesco.muoio@cardiocentro.org (F.M.); stefano.panella@cardiocentro.org (S.P.); 2 Institute of Chemistry & Biotechnology, Competence Center of Biochemical Engineering & Cell Cultivation Technique Zurich University of Applied Sciences, 8820 Wädenswil, Switzerland; jose@zhaw.ch (V.J.); eibs@zhaw.ch (R.E.); 3 Sferalp SA, 6998 Monteggio, Switzerland; mlin@sferalp.com (M.L.); mmul@sferalp.com (M.M.); 4 Department of Plastic, Reconstructive and Aesthetic Surgery, EOC, 6900 Lugano, Switzerland; yves.harder@eoc.ch; 5 Faculty of Biomedical Sciences, Università della Svizzera Italiana, 6900 Lugano, Switzerland

**Keywords:** serum- and xeno-free cell culture medium, *UrSuppe*, biodegradable microcarrier (MC), biomaterial, human adipose stem cells (hASCs)

## Abstract

Human adipose stem cells (hASCs) are promising candidates for cell-based therapies, but they need to be efficiently expanded in vitro as they cannot be harvested in sufficient quantities. Recently, dynamic bioreactor systems operated with microcarriers achieved considerable high cell densities. Thus, they are a viable alternative to static planar cultivation systems to obtain high numbers of clinical-grade hASCs. Nevertheless, the production of considerable biomass in a short time must not be achieved to the detriment of the cells’ quality. To facilitate the scalable expansion of hASC, we have developed a new serum- and xeno-free medium (*UrSuppe*) and a biodegradable microcarrier (*BR44*). In this study, we investigated whether the culture of hASCs in defined serum-free conditions on microcarriers (3D) or on planar (2D) cell culture vessels may influence the expression of some marker genes linked with the immature degree or the differentiated status of the cells. Furthermore, we investigated whether the biomaterials, which form our biodegradable MCs, may affect cell behavior and differentiation. The results confirmed that the quality and the undifferentiated status of the hASCs are very well preserved when they grow on *BR44* MCs in defined serum-free conditions. Indeed, the ASCs showed a gene expression profile more compatible with an undifferentiated status than the same cells grown under standard planar conditions.

## 1. Introduction

Adipose tissue is remarkably understudied compared with other organs. However, in recent years, due to increasing obesity and obesity-associated pathologies globally, this tissue has gained significant attention from the research community. Now it is recognized as a proper active endocrine organ at the center of energy balance and nutritional homeostasis, mediating the regulation of multiple organs and tissue [1,2,3]. Adipose tissue’s developmental biology is beginning to be understood and has proved surprisingly complex [4]. These studies have led to the discovery of several genes and proteins that regulate adipogenesis that can now be used to assess the status of hASCs in culture.

The adipose tissue has been recently identified as a novel abundant source of adult stem cells. A relatively noninvasive procedure, such as lipo-aspiration, gains access to the source tissue [5]. However, while being a rich source of stem cells, lipoaspirates presently cannot provide enough cells for direct applications in regenerative medicine [6]. Several clinical studies with human adipose stem cells (hASCs) are very promising and have reported that hASCs exerted beneficial effects in treating different disorders [7]. Human ASCs are increasingly in demand in plastic and reconstructive surgical procedures, where we are witnessing a shift toward tissue-engineering strategies with stem cells [8]. Currently available reconstructive surgery employing synthetic materials or autologous fat transplants is often disappointing due to volume maintenance’s long-term unpredictability. Hence, the clinical use of hASC has spread throughout the world. Consequently, this demand requires developing good manufacturing practices (GMP)-compliant ex vivo expansion protocols to sustain the supply of safe and cost-effective clinical-grade cells.

Several published studies have recently shown that traditional, planar, and static cultivation systems are not convenient to reach the high cell numbers required to implement cell-based therapies. Therefore, at the moment, the most promising alternative to overcome the drawbacks of planar static cultivation technologies are instrumented, stirred bioreactors in combination with microcarriers (MCs) [9]. These are microspheres (with a diameter of 100–300 µm) composed of various materials, encompassing synthetic/organic or natural polymers, manufactured with varying porosities and topographies. They furnish a surface upon which anchorage-dependent cells can attach and subsequently grow in cell culture medium by slow agitation [10].

When culturing stem cells in vitro, the amplification without affecting their “stemness” and differentiation potentials is essential. Thus, the most obvious solution would be to replicate the stem cell niche accurately. Unfortunately, reproducing this environment has not been possible because most of the mechanisms ruling the stem cell fate in vivo are yet not fully understood. Therefore, the cells are set to a “new” environment, defined by two main components, which we can modify and adapt to their needs: one, the cell culture medium and conditions, and two, the substrate where the hASCs grow. This “new” environment might endanger the control of the cultured stem cells’ fate.

To overcome these hazards and ensure control over cell fate, we developed a defined xeno- and serum-free medium, called “*UrSuppe*”, specially engineered to grow hASCs. Fetal bovine serum (FBS) is still one of the prevalent ancillary materials used in cell culture, although it is an incompletely characterized blend containing numerous growth factors and hormones. Therefore, it is tough to control the stemness of the expanded hASCs in this component’s presence [11]. Other, more classical, considerations apply to avoid FBS in the cell culture medium used to grow therapeutic hASCs. Indeed, when FBS is used, the risk of transferring zoonoses cannot be excluded. Furthermore, fetal bovine proteins are internalized by the stem cells or stick on their surface, inducing an immune xenogenic rejection upon autologous transplantation into the human patient [12]. Finally, except for blood and endothelium, all the other cells in the tissues are usually never in direct contact with serum. They are immersed or surrounded by the interstitial fluid, which has a lower concentration of proteins than serum, and it is often distinct for each tissue or organ due to a unique blend of secreted polypeptides and metabolic molecules. This is a situation that we try to recreate and mimic in defined serum-free conditions. The same applies to the platelet lysate (PL): despite being produced from human blood, it is a cocktail of numerous growth factors and hormones, some of which with a well-known adipogenic promoting activity, which acts in an unpredictable and uncontrolled way on the fate of the hASCs [13].

The control of stem cell fate, either in vivo or in vitro, has been ascribed mainly to molecular or biochemical mediators, such as hormones, growth factors, or interactions of extracellular matrix (ECM) ligands with cell surface receptors [14]. A variety of physical signals from the environment also contribute to the cell stemness’ overall control. More specifically, it is now known that the ECM geometry/topography at the nano-scale range, the ECM biomechanical properties (e.g., elasticity versus stiffness), and the transmission of mechanical (e.g., shear stress) or other biophysical factors to the (stem) cells may strongly influence their fate [15]. Therefore, the substrate where the hASCs grow is the second principal component that we can fine-tune to transmit the appropriate cues to the cells and represent a significant scientific challenge. We need to reproduce with biomaterials the complexity of the ECM.

Currently, expansion protocols utilize planar static monolayer cell expansion in flask or cell factories, but these systems are limited since they cannot reach hundreds of billions of cells. However, in the field of regenerative medicine, this could barely satisfy one treatment dose, and a patient may require multiple doses [16,17].

With this in mind, we also developed a new biodegradable microcarrier, named *BR44*, tailored for the chemically defined cultivation of hASCs in dynamic systems. Biodegradable MCs are advantageous as it is unnecessary to harvest the cells or use new scaffolds for cell transfer. High cell viability and potency could be easily preserved with this MC type, simplifying the downstream processing steps (DSP) when manufacturing therapeutic hASCs. The criteria necessary to assemble a useful scaffold that mimics the biological microenvironment (the cell “niche”) have not yet been fully understood. Therefore, the lack of specific guidelines imposed to empirically verify each new scaffold’s suitability combined with cells and the culture medium [18]. Therefore, in this study, we wanted to verify whether hASCs growing on the biodegradable MC *BR44* or the commercial non-biodegradable MC ProNectin-F (PNF, Pall Solohill) preserve or not their undifferentiated status.

## 2. Materials and Methods

### 2.1. Microcarriers Production

The production of the microcarriers was extensively described in Muoio et al. [19]. Briefly, the manufacturing process relies on a double emulsion solvent evaporation technique where the most critical components for the manufacture are 17 KDa Poly(D, L-lactide-co-glycolide) (PLGA) (lactide/glycolide ratio 50:50), Purasorb PDLG 5002 purchased from Corbion Purac (Amsterdam, Netherlands). Polyvinyl alcohol (PVA, 87–89% hydrolyzed, Mw 31 KDa) and methylene chloride from Merck (Darmstadt, Germany). Finally, porcine gelatin, a generous gift from Italgelatine (Santa Vittoria d’Alba, Italy), and ammonium hydrogen carbonate from Applichem (Darmstadt, Germany).

### 2.2. Extraction of Adipo-Cutaneous Tissue

The donors of the subcutaneous adipose tissue used in this study provided written agreement as required from the local Ethics Committee of the Canton of Ticino, which approved the project and its procedures (project reference number: CE 2915). The surgical removal of adipose tissue was described by Panella et al. [19]. Adipose tissue samples were stored at room temperature and processed at the latest 24 h after their collection [20].

### 2.3. Isolation and Culture of hASCs

#### 2.3.1. Isolation of Stromal Vascular Fraction

Isolation of the cells of the SVF from human adipose tissue, in vitro culturing in defined xeno- and serum-free conditions, cell cryopreservation, followed the ethical principles traced in the Declaration of Helsinki and according to the instructions of the Ethics Committee of the Canton of Ticino (Switzerland). The protocol we routinely use to extract the SVF is described precisely by Panella et al. [19]. We briefly summarize the essential parameters here: the adipose tissue was homogenized in a blender then digested for 45 min at 37 °C with 0.28 Wünsch Unit/mL of Collagenase AB (Worthington Biochemical Corp., Lakewood, NJ, USA). The addition of DPBS supplemented with 5% injectable human albumin CSL (Behring AG, Bern, Switzerland) facilitated the separation of the aqueous from the lipid phase. The aqueous phase cells were collected, filtered through sieve cell strainers, and pelleted by centrifugation at 600× *g* for 10 min at room temperature. Finally, the cell pellet was resuspended in DPBS with 1% injectable human albumin or in the tissue culture medium.

#### 2.3.2. Characterization of SVF Cells by Flow Cytometry

Freshly isolated cells of the SVF were stained for analytical flow cytometry as described by Panella et al. [19]. Briefly, the cells were incubated 20 min with the following monoclonal mouse anti-human antibodies and fluorescent dyes: CD34-BV650, CD45-PC7, CD73-FITC (BioLegend, San Diego, CA, USA), CD146-PE, CD36-APC (Miltenyi BioTech, Bergisch, Germany), 7-amino-actinomycin D (7-AAD) (Becton Dickinson, Franklin Lakes, NJ, USA), and Syto40 (Life Technologies, Thermo Fisher Scientific, Waltham, MA, USA). After that, the erythrocytes were lysed with 200 μL of VersaLyse solution (Beckman Coulter Inc., Pasadena, CA, USA). ASCs are described as CD45-, CD146-, CD36-, CD34+, and CD73+ cell populations. Samples were acquired according to our flow cytometer procedure. More details about this protocol can be found in the Supplementary Materials Table S1, which accompanies and complements this study.

#### 2.3.3. Flow Cytometer Procedure

The CytoFLEX Flow Cytometer was used to acquire the raw data analyzed using the Kaluza software (both from Beckman Coulter Inc., Pasadena, CA, USA). Spill-over coefficients were established employing single-stained control particles (VersaComp Antibody Capture Bead Kit, Beckman Coulter) or appropriately single-stained cells. The compensation matrix was worked out using the dedicated function of the Kaluza software. Optical alignment and fluidics were periodically monitored using flow-check fluorospheres (CytoFLEX Daily QC Fluorosphere, Beckman Coulter, Brea, CA, USA).

#### 2.3.4. Initial Seeding of the SVF Cells and Cell Passaging

After the adipose tissue extraction, hASCs were cultured right away only in our proprietary defined serum- and xeno-free cell culture medium called *UrSuppe.* This latter was developed to culture hASCs and could be produced under GMP conditions. It contains exclusively pure molecules, some recombinant human growth factors, and injectable human albumin in the µg range. After the characterization of SVF cells by flow cytometry, hASCs were plated at a density of 30,000–50,000 hASCs/cm^2^ in fibronectin-peptide pre-coated cell culture vessels (Corning PureCoat™ ECM Mimetic Fibronectin Peptide, Corning Inc., Corning, NY, USA). The medium was replaced every 2 days, keeping 50% of it, until the cells were between 80% and 90% confluent. In the Supplementary Information of Panella et al. [19], we describe in detail the initial plating of the cells (Section 3), and we give some suggestions on how to work in defined serum-free conditions (Section 4). For passaging, the cells were detached with TrypLE^TM^ Select (Life Technologies, Thermo Fisher Scientific, Waltham, MA, USA) after 2 min of incubation at 37 °C. The hASCs were washed with DPBS at least once to remove the protease, counted, resuspended in *UrSuppe* medium, and finally seeded in new cell culture vessels (Corning PureCoat™ ECM Mimetic Fibronectin Peptide, Corning Inc., Corning, NY, USA) at a density of 10,000 hASCs/cm^2^.

### 2.4. Cells Seeding on MCs for Static Experiments

Protocol for efficient seeding of hASCs on the microcarriers we used for the experiments: (A) Cell preparation procedure: After detachment, the cells were washed with DPBS supplemented with 1% human albumin, counted with Trypan Blue (Life Technologies, Thermo Fisher Scientific, Waltham, MA, USA), and resuspended in *UrSuppe* medium at a concentration of 280,000 cells/µL. (B) Preparation of the positive control MC: The lyophilized PNF MCs (Pall SoloHill, Port Washington, NY, USA) were incubated in *UrSuppe* medium for 1 h at 37 °C in agitation. (C) Preparation of the test MCs: The *BR44* prototype manufactured by Sferalp SA (Monteggio, Switzerland) was washed in DPBS and then incubated in *UrSuppe* for 1 h at 37 °C in agitation and finally washed with DPBS again. Just before the tests, the MCs were resuspended in 250 µL *UrSuppe*. The surface area available for cell adhesion was 2.5 cm^2^ (PNF) or 1 cm^2^ (*BR44*). Adhesion and proliferation assays of hASCs growing on MCs were performed in vessels developed to avoid the cells’ attachment to the plate surface (Ultra-Low Attachment surface, Corning Inc., Corning, NY, USA). A total of 250 µL of MCs suspension were seeded in each well and gently mixed to distribute the carriers. Then, 250 µL of ASCs suspension were added, drop by drop. After gently mixing, the plate was put in the incubator (37 °C, 5% CO_2_), and the hASCs were cultured for 7 days. Every 2 days, half of the medium was removed and replaced with a fresh one.

### 2.5. Attachment and Growth of hASCs in Static Conditions

#### 2.5.1. Cell Adhesion—Nuclei Analysis

Nuclei staining is a reliable approach to assess cell density during the microcarriers-based culture of adherent cells. After 2–4 days of growth, the carrier-cell aggregates were fixed (30 min on ice) with a DPBS solution containing 2% formaldehyde and 0.2% glutaraldehyde (both from Sigma-Aldrich Inc., St. Louis, MO, USA). The samples were washed two times with DPBS, resuspended in DPBS containing 500 nM DAPI (Sigma-Aldrich Inc., St. Louis, MO, USA), and finally incubated for 20 min in the dark at room temperature. After that, the samples were washed twice with DPBS, and the carrier-cell aggregates were resuspended in 85% glycerol solution and mounted for microscope inspection. Images were acquired with a UV laser (405 nm) on an inverted fluorescence microscopy Nikon Eclipse Ti (Nikon, Minato, Tokyo, Japan).

#### 2.5.2. Cells Adhesion—Scanning Electron Microscope (SEM) Analysis

After 2–4 days of growth on the MCs, the carrier-cell aggregates were fixed with a 3% glutaraldehyde solution for 30 min on ice. Next, the aggregates were washed twice with DPBS, resuspended in distilled H_2_O, and let completely dry on a metal stub at RT for 3 h. Finally, the samples were coated with gold/palladium in a Mini Sputter Coater at 18 mA for 60 s and analyzed with a TM3030 Hitachi microscope (Tokyo, Japan) at 15 kV.

#### 2.5.3. Analysis of Nuclei Released from hASCs Grown on MCs by Flow Cytometry

The proliferative status of hASCs grown on MCs was investigated by analyzing samples taken on different days. The full protocol for the nuclei extraction from hASCs grown on MCs is reported in the Supplementary Materials that accompanies and completes this article, paragraph “*Evaluation of cell proliferation*” and Figure S1. After the extraction, the nuclei were stained with 1.25 ng/µL of 7-AAD and analyzed by flow cytometry using the CytoFLEX Flow Cytometer manufactured by Beckman Coulter Inc. The acquired data were elaborated with the Kaluza software (Beckman Coulter Inc., Brea, CA, USA). The gating strategy to exclude debris and aggregates has been explained in Muoio et al. [21] in the Supplementary Materials Figures S8 and S9.

### 2.6. Evaluation of Cellular Phenotype

#### 2.6.1. Cell Detachment and Harvesting from the MCs for Flow Cytometry or RT-qPCR Analysis

To detach the hASCs from the MCs, the carrier-cell aggregates were transferred into a 15 mL tube and digested for 10 min at 37 °C with TrypLE^TM^ Select (ThermoFisher Scientific, Waltham, MA, USA) by gentle agitation. After washing away the protease with DPBS/1% injectable human albumin, the hASCs were separated from the MCs by filtering the mixture through a pluriStrainer Mini 40 µm mesh sieve (PluriSelect life science, Leipzig, Germany). The cell suspension was centrifuged at 400× *g* for 5 min. The pellet was resuspended in FACS buffer (DPBS supplemented with 1% injectable human albumin and 50 ng/µL human immunoglobulins, Privigen Immunoglobulin, CSL Behring AG, Bern, Switzerland) for flow cytometry analysis, or left as a pellet for total RNA extraction and RT-qPCR analysis.

#### 2.6.2. Flow Cytometry Analysis of hASCs Grown on Cell Culture Vessels or MCs

Each assay contained a unique combination of the following mouse anti-human monoclonal antibodies: CD73-FITC, CD90-APC, CD105-PE (BioLegend, San Diego, CA, USA), CD34-PE, CD36-APC, CD146-PE (Miltenyi BioTech, Bergisch, Germany), CD61-PE (Beckman Coulter Inc., Pasadena, CA, USA), CD15-FITC, and 7-AAD (Becton Dickinson, Franklin Lakes, NJ, USA). All antibodies were titrated and used at a 50 ng/assay concentration. Isotype controls and specific mAbs were employed at the same final concentrations. Routinely, 50,000 hASCs (in 100 µL FACS buffer) were mixed with the appropriated antibody combination and incubated for 15 min at RT in the dark. Finally, the sample was diluted with 100 µL FACS buffer before the acquisition, according to our flow cytometer procedure (see Section 2.3.3).

#### 2.6.3. RT-qPCR Analysis

According to the manufacturer’s instructions, RNAs were extracted from cell pellets or carrier-cell aggregates employing the Nucleospin^®^RNA kit (Macherey-Nagel, Düren, Germany). The kit’s purification procedure foresees an on-column digestion step with DNase I. RNA purity and quantity were evaluated with the NanoDrop device (Thermo Fisher Scientific, Waltham, MA, USA). Total RNA integrity was periodically checked by conventional agarose gel electrophoresis. Typically, 400 ng of RNA were transcribed into cDNA by the reverse transcription-polymerase chain reaction performed by the commercial kit “GoScript™ Reverse Transcription System” (Promega, Madison, WI, USA). This reaction is summarized in the Supplementary Materials Table S2. The genes *SOX9*, *RUNX2*, *PPARG*, *PREF1*, *ZFP423*, *ZFP521,* and *DKK1* were subjected to RT-qPCR using the “SsoAdvanced Universal SYBR^®^ Green Supermix” kit (Biorad, Hercules, CA, USA), and the results detected with the “CFX Connect Real-Time PCR Detection System” (Biorad, Hercules, CA, USA). For each gene under investigation, RT-qPCR was performed with 20 ng of cDNA. *ACTB* was employed as an internal control. Each primer pair product was checked for proper amplification by agarose gel electrophoresis. We used exclusively primer pairs, which gave rise to single sharp bands of the expected size. Primer sequences, temperatures, and cycle conditions are shown in the Supplementary Materials Tables S3 and S4. Data were analyzed with the CFX software to calculate the relative fold gene expression using the formula 2^−ΔΔCt^.

### 2.7. Proof-of-Concept Cultivation in Spinner Flasks

Proof-of-concept cultivation was performed for one donor (*n* = 1) with the *BR44* MC in a disposable Corning spinner flask (working volume = 100 mL). The spinner flask was inoculated with an initial cell density of 20,000 cells/cm^2^ (=38,794 cells/mL) and with 5 g/L (≈194 cm^2^) of the *BR44* MC. The MC concentration and cell-to-bead ratio for the inoculation were defined based on previous cell attachment studies performed in well plates (data not shown). The cell inoculum was prepared in T75-flasks coated with Synthemax II coating (Corning Inc.) and with cells from P1. Before inoculation, the MCs were equilibrated in the *UrSuppe* cell culture medium for up to 12 h. A static cell attachment phase was performed for 24 h in a cell culture incubator (37 °C, 5% CO_2_, 80% rH). After the cell attachment, the cell culture was continuously stirred at 55 rpm. A partial medium exchange of 30% (no MC feeds) was performed on day 5. Offline samples were taken daily to measure substrate and metabolite concentrations (Glc, Lac, Amn, San Diego, CA, USA) with a Cedex Bio (Roche Diagnostics, Rotkreuz, Switzerland). The hASC cell number was measured using a NucleoCounter NC-200 (ChemoMetec, Allerod, Denmark) combined with the Via-1-cassette. In addition to the cell measurements, 1 mL of the MC-cell suspension was fixed after sampling with a 3% PFA solution for DAPI staining. Based on the cell-specific values, growth-related parameters were calculated according to the equations indicated in the Supplementary Materials; see the paragraph “*Evaluation of growth-related parameters*”.

### 2.8. Biochemical Analysis

#### 2.8.1. Proteome Profiler Array

The conditioned medium from cell grown in planar, static 3D, and 3D dynamic conditions, were investigated to monitor selected secreted polypeptides. Human ASCs of the three tested cell culture systems were left with the same medium for 7 days before collecting the supernatants. These latter were initially filtered through 0.22-micron Syringe Filters (Jet Biofil, Guangzhou, China) to remove the particulate. Next, 1 mL of each supernatant was poured onto a nitrocellulose membrane containing many different capture antibodies and left overnight by gentle rocking. According to the instructions provided with the “Proteome Profiler Human Adipokine Array Kit” (R&D Systems, Minneapolis, MN, USA), all subsequent manipulations were undertaken. The secondary biotinylated detection antibodies were highlighted by the use of Streptavidin conjugated IRDye 800 CW (Li-Cor Corporate, Lincoln, NE, USA). Positive signals were detected after scanning the membrane with an Odyssey^®^CLX Imaging System (Li-Cor Corporate, Lincoln, NE, USA). Data were elaborated using Li-Cor software to evaluate the signal intensity ratio between the different adipokine spots and the positive control reference spot.

#### 2.8.2. Amino Acid Analysis

The conditioned media from cells grown in standard planar 2D, static 3D, and dynamic 3D conditions were analyzed according to Bidlingmayer et al. [22] to determine the amino acid concentration in the supernatants. *Coupling reaction*: 10 μL of each sample were pipetted in a glass tube (5 × 60 mm) with 5 μL EDTA-solution and dried on a vacuum concentrator (Speedvac). Then 10 μL of Coupling Solution A (MeOH/H_2_O/Triethylamine (2:2:1)) were added and dried again on a vacuum concentrator. The samples were redissolved in 10 μL Coupling Solution B (MeOH/H_2_O/Triethylamine/Phenylisothiocyanate (70:10:10:1)), incubated for 20 min at RT, and dried for 30–45 min. *Standard*: Reference solutions of L-Alanyl-L-Glutamine, L-Glutamine, Trans-4-Hydroxy-L-Proline, and L-Tryptophane, were prepared at a concentration of 2.5 µmol/µL in 0.1% HCl. To 50 µL of the amino acid standard H (Thermo Scientific^TM^ Pierce^TM^, 2.5 µmol/µL each), 50 µL of each amino acid reference solution were added and diluted to 1 µL with 0.1 M HCl (=125 nmol/µL each). Then, 10 µL of this standard solution was treated as described before (see Coupling reaction). *Analysis*: The samples were reconstituted in 50 μL ammonium acetate buffer pH 6.4 containing 4% acetonitrile (HPLC eluent [A]). Injection volume: 20 μL; Standard = 500 pmol/AS (except Cystine = 250 pmol). HPLC: Column: Waters Nova-Pak C18, 4 mm, 60 A, 3.9 × 125 mm. Device: Thermo Ultimate-3000, UV detection at 247 nm, and column temperature 50 °C. Eluent A: 96% 0.14 M Ammonium acetate, 575 µL Triethylamine PH 6.4. Eluent B: 40% H_2_O Milli-Q, 60% Acetonitrile. Gradient: 3% [B] to 50% [B] in 12 min.

### 2.9. Statistical Analysis

Statistical analyses were performed using SPSS (IBM, Armonk, NY, USA) or Prism 7 (Graph Pad Software Inc., San Diego, CA, USA). The assumption of the analysis was checked: normal distribution and homogeneity of the variance. *p*-values lower than 0.05 were considered statistically significant. The differences between groups for cellular growth on the MCs were evaluated with a two-way repeated-measures ANOVA. The differences between groups for flow cytometry and gene expression profile were evaluated with a one-way ANOVA.

A list of the materials used in this study, which complements the information given in this section, can be found in the Supplementary Materials Table S5.

## 3. Results

### 3.1. Properties and Stability of Microcarriers

**Figure 1:**
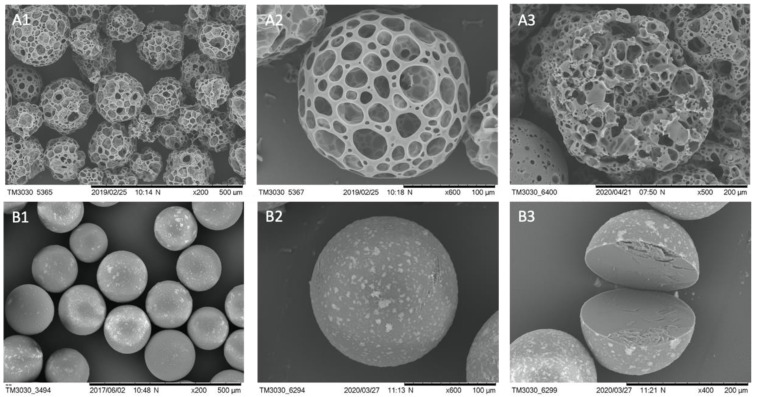
SEM microphotographs to show the morphology of the *BR44* and PNF MCs. (**A**) *BR44*: (**1**) 200×, scale bar 500 µm (**2**) 600×, scale bar 100 µm (**3**) 500×, scale bar 200 µm (broken MCs). (**B**) PNF: (**1**) 200×, scale bar 500 µm (**2**) 600×, scale bar 100 µm (**3**) 400×, scale bar 200 µm (broken MCs).

The *BR44* manufacturing process consists of a double emulsion, solvent evaporation system, generating a blend of poly lactic-co-glycolic acid (PLGA) and porcine gelatin. Scanning electron microscopy (SEM) of the *BR44* showed an excellent regularity of this MC’s shapes and size based on resorbable and biodegradable biopolymers. Structurally, the microcarrier has a spherical shape, reticulated frame, and a very porous consistency, also internally (Figure 1(A1–A3)). We used the ProNectin F (PNF) MC as a positive control for our tests. This bead performs well in defined serum-free media, like the *UrSuppe* cell culture medium [23]. PNF MC consists of a synthetic core bead (polystyrene) with a thin layer of recombinant, non-animal source polymer on its surface that incorporates multiple copies of the RGD attachment signal sequence of the human fibronectin protein. The microphotographs show a very regular shape and size, with internally a solid and compact construction (Figure 1(B1–B3)). However, the coating is not evenly distributed on the carriers’ surface but instead with a spotted pattern (Figure 1B). On the other hand, the gelatin coating of *BR44* MC is homogeneously distributed inside and on the microsphere’s surface, as shown by Muoio et al. [21].

**Figure 2:**
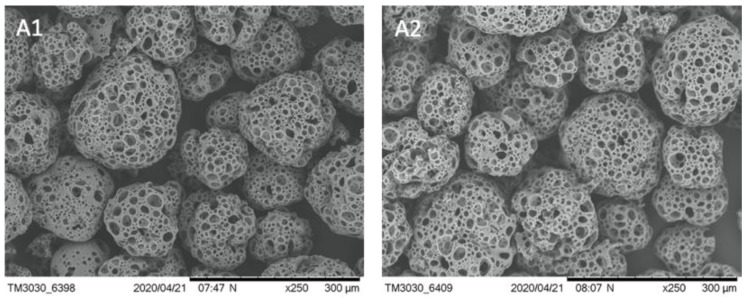
SEM microphotographs to show the stability of *BR44* MC at 4 °C in water. (**A1**) *BR44* newly produced: (**A2**) *BR44* after 2 months in water at 4 °C. (250×, scale bar 300 µm).

**Figure 3:**
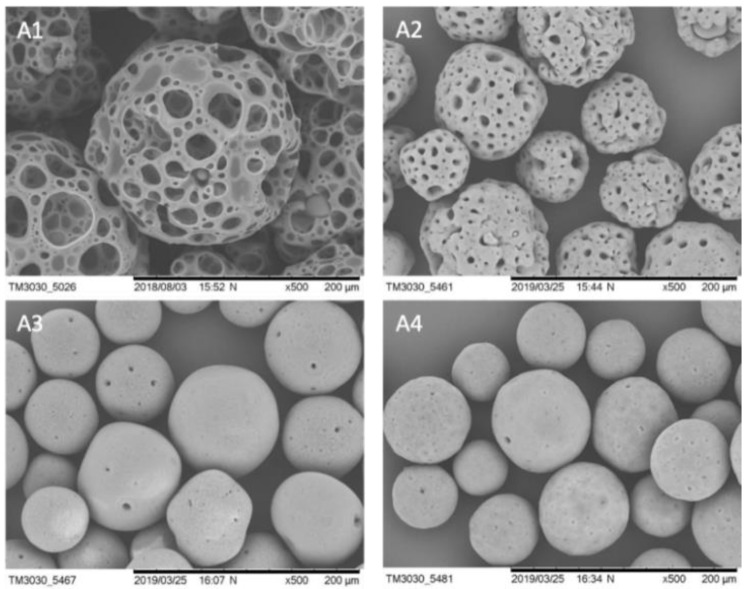
SEM microphotographs to show the erodibility of *BR44* MC at 37 °C in cell culture medium *UrSuppe* and under stirred conditions. (**A1**) *BR44* on day 0: (**A2**) *BR44* on day 1, (**A3**) *BR44* on day 4, (**A4**) *BR44* on day 9. (500×, scale bar 200 µm).

We checked the stability of *BR44* MC in an aqueous solution. It is essential to verify that the microspheres are stable at 4 °C for a significant time. At the same time, biodegradability should occur at 37 °C, with the usual parameters used to cultivate cells. We found that the *BR44* was stable in water at 4 °C for up to 2 months, and no signs of degradation were observed (Figure 2). The biodegradability and other bioengineering parameters (size variation/shape variation and sedimentation velocity) of *BR44* MC were evaluated and described by Muoio et al. [21]. Figure 3 shows the change in the shape of *BR44* MCs under stirred conditions at 37 °C after 1, 4, and 9 days, respectively, confirming the carrier’s erodibility.

### 3.2. Static Investigations: Cell Adhesion and Proliferation on MCs

**Figure 4:**
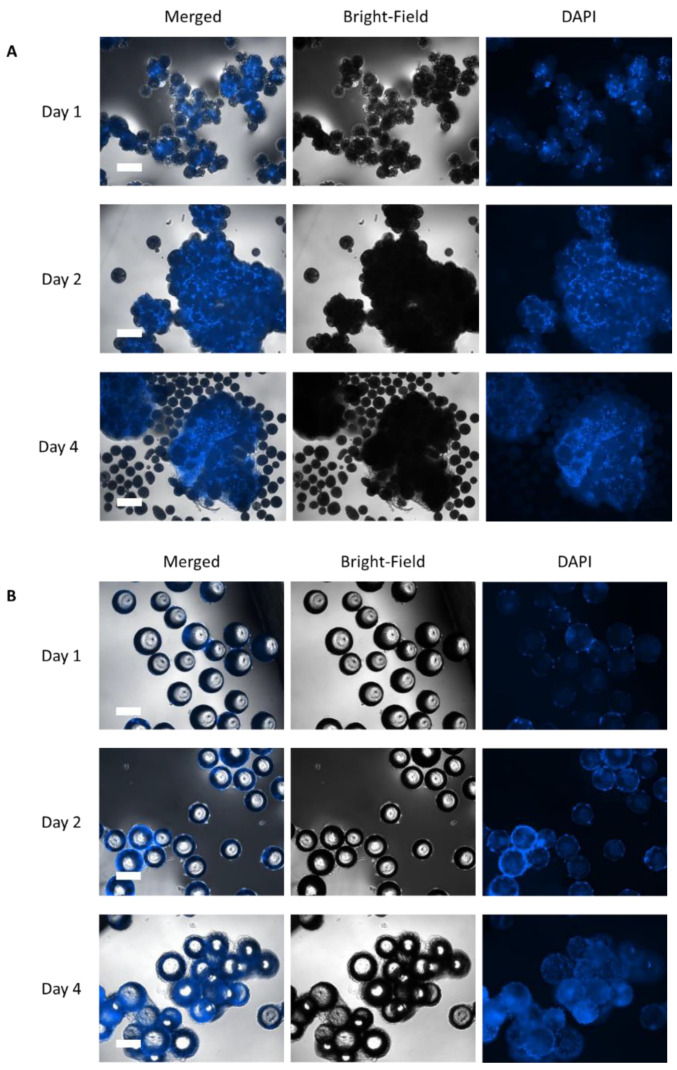
DAPI stained samples of hASCs cultured on MCs in a static condition after 1, 2, and 4 days of culture. (**A**) *BR44* MCs, 100×, scale bar 300 µm (**B**) PNF MCs, magnification: 200×, scale bar 150 µm.

**Figure 5:**
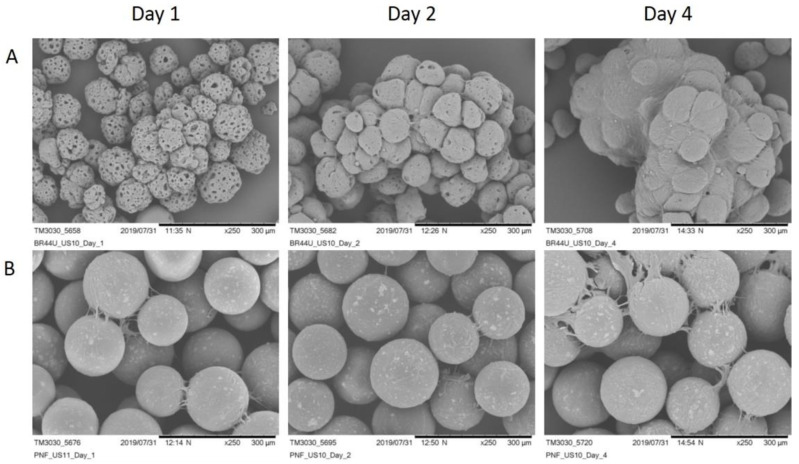
SEM microphotographs to confirm the presence of hASCs on MCs. *BR44* MCs (**A**) and PNF MCs (**B**) after 1, 2, and 4 days of static culture. Fold magnification: 250×, scale bar 300 µm.

MC prototypes were manufactured and evaluated for their capacity to permit the adhesion of hASCs under serum- and xeno-free conditions. We found that the *BR44* MC prototype promoted cell attachment and proliferation comparable to a commercial product, PNF MC. Figure 4 and Figure 5 show the efficient attachment and growth hASCs on the *BR44* MC compared to the PNF MC.

After few days of culture in static conditions, the MCs are completely colonized by cells. We observed large aggregates’ formation confirming the suitability of *BR44* as a three-dimensional scaffold to grow primary cells in serum and xeno-free conditions. PNF MC also performed very well, and the cells colonized it quickly, efficiently, and entirely as shown in Figure 4B. However, we noticed that with PNF MC, the formed aggregates’ size is smaller than that observed when using *BR44* MC. Thus, for reasons currently unknown, the *BR44* MC-based culture system appears to be “stickier” than that dependent on PNF MC.

**Figure 6:**
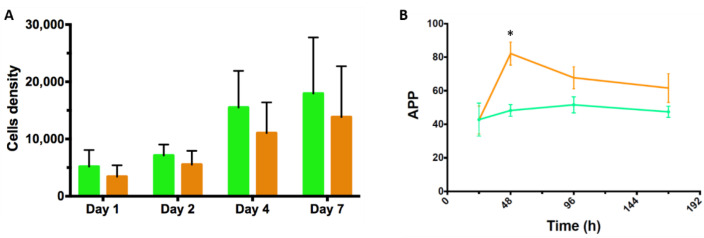
Analysis of 7-AAD stained nuclei released from MCs-based cultures. The nuclei can be divided into three categories: nuclei with a DNA content of 2N (G_0_ and G_1_ phases), those with a DNA quantity corresponding to 4N (G_2_ phase), and finally, those with an intermediate DNA content (S phase). The three phases of interest, G_1_, G_2_, and S, are distinguishable by flow cytometry. (**A**) Volumetric flow cytometry was employed to establish the number of nuclei released from the carrier-cells aggregates. Graphical representation of the nuclei counts of three patients hASCs’ grown on *BR44* MC (green bar) and PNF MC (orange bar). The data represent cell densities [cells/cm^2^] and are shown in function of four measurements (days 1, 2, 4, and 7), (*n* = 3, error bars represent S.E.M.). No significant differences were observed between cells cultured on *BR44* or PNF microcarriers. (**B**) Graphical representation of the active proliferation percentage (APP): the percentages of nuclei found in G_1_, G_2_, or in the S phase were determined for hASCs grown on *BR44* MC and PNF MC. A slightly significant difference in APP was founded only on day 2 (*p* = 0.042). The measurements were done after days 1, 2, 4, 7, and APP percentage was calculated as follows: APP = [(G_2_ + S)/G_1_] × 100. (*n* = 3, error bars represent S.E.M.).

It is often difficult to detach with high yield cells from the MCs they have grown on. For this reason, we developed a procedure based on nuclei count: A cell lysis buffer solution frees the nuclei that can be enumerated by flow cytometry. This method also allows assessing the cell cycle status of the collected nuclei. Therefore, it is also possible to determine the proliferative status of the hASCs grown on the *BR44* MC or PNF MC (for more information, see the “Materials and Methods” section, Section 2.5.3 “Analysis of nuclei released from hASCs grown on MCs by flow cytometry”). As shown in Figure 6, although the *BR44* MC prototype does not usually reach active proliferation rates greater than or equal to PNF, the results obtained, compared to the “top of the class,” are all very remarkable. Thus, we can conclude that *BR44* MC allows hASCs to adhere on its surface and maintains a significant percentage of cells in active proliferation in static conditions during the 7 days analyzed in these experiments.

### 3.3. Flow Cytometry Analysis of Some Standard Markers in Static Conditions

**Figure 7:**
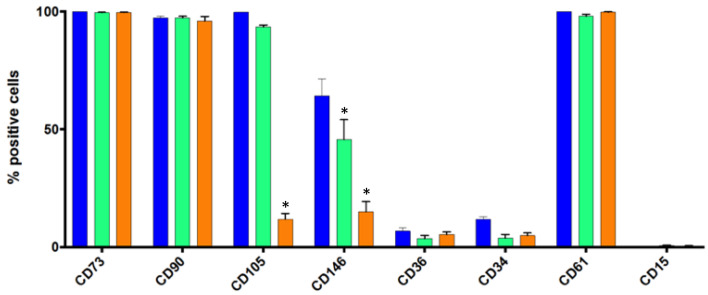
Percentages of primary hASCs positive for the eight cell surface markers picked out for this analysis. Bar chart recapitulates flow cytometry average of data obtained from three different donors of hASCs. Cell culture condition: blue bar, standard 2D cell culture vessels; green bar, Figure 3. D condition with *BR44* MCs; orange bar, defined static 3D with PNF MC. The cell culture medium used: *UrSuppe*. (*n* = 3, error bars represent S.E.M.). The single-parameter histograms are shown in the Supplementary Materials, Figures S3–S6. * Reduction of expression compared to 2D culture are statistically significant: *p*-value < 0.0001.

It is very important to certify that the hASCs growing on *BR44* MC (static 3D conditions) exhibit the same standard surface markers and preserve an immature status as the same batch of cells cultivated in traditional cell culture vessels (2D conditions). After 7 days of culture, flow cytometry analysis of these surface proteins highlighted some notable distinctions between cells grown in 2D from those grown in 3D on *BR44* MC or PNF MC (see Figure 7). The canonical markers [5] CD73 and CD90 showed no differences in the three conditions tested. However, with PNF MC, the number of CD105 positive hASCs was lower (statistically significant result). A second substantial difference concerned the CD146 marker, whose expression decreased on MCs. This cell surface marker protein is associated with adipogenic differentiation. Indeed, Graham G. Walmsley et al. showed that hASCs induced to differentiate into adipocytes expressed the CD146 as well as the CD36 markers [24]. Therefore, these observations lead us to consider as a positive factor the decrease in the number of positive CD146 hASCs observed when the cells were cultured on MCs (statistically significant result). The most significant decrease was observed in combination with the PNF MC. This suggests that the type of MC used for the culture can shape the cells’ phenotype.

CD61 is a control marker related to the cells’ undifferentiated status and is not expressed by human fibroblasts [25]. The cells we analyzed are practically 100% positive for this marker, so the cultures do not appear to be contaminated with fibroblasts. The low percentage of CD15^+^ or CD34^+^ is an excellent feature of the hASCs grown in *UrSuppe* medium, which signalize being mostly immature cells. Taken together, the hASCs grown on MCs do not degenerate and maintain their undifferentiated status, at least as well as those cultured in standard cell culture vessels. This consideration is also supported by the low percentage of CD36 positive cells, an established marker for ongoing adipogenesis [26,27,28].

### 3.4. Proof-of-Concept Cultivation in Small Scale Spinner Flasks

**Figure 8:**
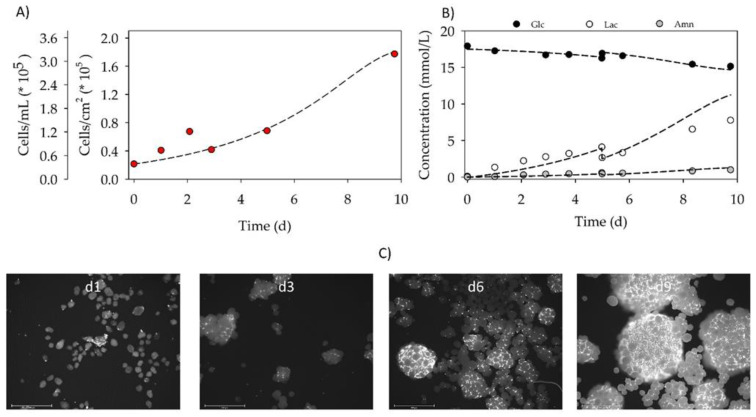
Time-dependent profiles of cell density (**A**), substrate/metabolite concentrations (**B**), and DAPI stained microscopic pictures during the proof-of-concept cultivation in the spinner flask (**C**). A partial medium exchange of 30% was performed on day 5. The symbols represent the experimentally measured values collected from offline measurements. The lines represent the simulated time courses, which were calculated according to [23]. Scale bar for fluorescence microscopic pictures = 650 μm.

Figure 8A,B shows the time-dependent profiles of the cell density and the substrate/metabolite concentrations during the proof-of-concept cultivation in a spinner flask with the *BR44* MC. After 24 h cell attachment phase, an attachment efficiency of 188% was achieved. This result indicates that a significant number of the cell population had already started to divide within the static cell attachment phase. A peak cell density of 1.78 × 10^5^ hASCs/cm^2^ (=3.18 × 10^5^ hASCs/mL) was achieved on day 10. Due to the “natural” stickiness of biopolymers (e.g., gelatin), *BR44* MCs tend to aggregate early during the cultivation, which affected the sampling. A maximum expansion factor of 8.1 was achieved on day 10. The hASCs grew at a specific growth rate of 0.25 d^−1^ (->t_d_ = 67.2 h). Based on the calculated growth-dependent parameters, cell growth was simulated according to our unstructured, segregated growth model [23,29]. The deviations between the experimentally measured and simulated cell densities were mainly due to the stickiness of the *BR44* MC and the more difficult sampling. However, the proof-of-concept cultivation results demonstrate the growth of hASCs on *BR44* MCs under dynamic conditions. The growth parameters of *BR44* are summarized in Table 1.

During the cultivation, glucose concentration decreased to a minimum value of 15.15 mmol/L, demonstrating that glucose was not a limiting factor. As a result of glucose metabolism, lactate concentration increased to a maximum value of 7.77 mmol/L, which resulted in a Y_Lac/Glc_ of 2.6. This high Y_Lac/Glc_ value indicates that additional Lac might be released from the MCs during the cultivation because the maximum values of 2 for Y_Lac/Glc_ can be expected from the glycolysis pathway. The ammonia concentration remained at 0.99 mmol/L, relatively low during the entire cultivation. Consequently, both lactate and ammonia levels were below critical concentrations (Lac_crit_ ≈ 35 mM, Amn_crit_ ≈ 2.4 mM) described in the literature [10,30,31,32,33].

Figure 8C shows microscopic fluorescence pictures of DAPI-stained hASCs on *BR44* MC during the spinner flask cultivation. It is recognizable that the aggregate size increased as a function of the cell density. Already on day 3, the first MC-cell-aggregates were visible. On day 9, most of the MCs were part of MC-cell-aggregates, which were highly confluent.

### 3.5. Gene Expression Survey by RT-qPCR of Some Essential Factors Regulating Adipogenesis

**Figure 9:**
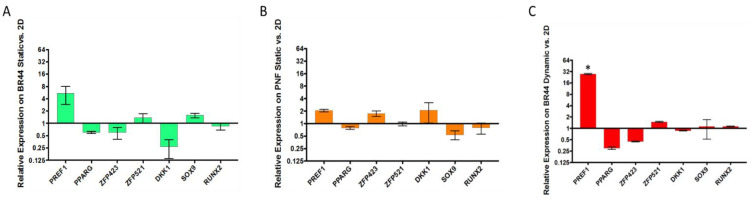
Relative expression levels of some genes regulating the adipocyte differentiation process measured by RT-qPCR. Primary hASCs from three different donors were grown in standard 2D cell culture vessels, in static 3D cell culture system on *BR44* MC (**A**), in static 3D cell culture system on PNF MC (**B**), and in dynamic 3D cell culture system on *BR44* (**C**). The calculated expression levels of the different markers for the 3D tests are related to the values obtained in the 2D conditions. (*n* = 3 error bars represent S.E.M.). * Statistically significant: *p*-value < 0.0001.

With these experiments, we wanted to verify whether the cell culture methods influenced the marker genes’ expression we selected for this assay. ASCs obtained from three different donors were cultured with *UrSuppe* medium in four separate ways: 1. In standard static cell culture vessels (2D). 2. On *BR44* MC in static conditions. 3. On PNF MC in static conditions. 4. On *BR44* MC in dynamic conditions. RT-qPCR was used to measure the expression level of the marker genes depicted in Figure S7. The normalized values obtained were related to those acquired from hASCs grown in static 2D conditions, and the final data visualized with three histograms, as shown in Figure 9A–C. Among the essential genes linked with the undifferentiated status of hASCs, there are *PREF1* [34], *ZFP521* [35], and *SOX9* [36], whereas *PPARG* [37], *ZFP423* [38], and *DKK1* [39,40] are known adipogenic commitment and lineage differentiation factors (for review see [41]). *RUNX2* [42] was included in the test panel to check whether the hASCs accidentally differentiated towards a chondrogenic or osteogenic lineage. As shown in Figure 9A–C, the relative expression levels of *PREF1*, the seal of the undifferentiated status of hASCs, were higher in all 3D conditions. However, only for the cells grown on *BR44* MC in dynamic conditions, the increase of *PREF1* was statistically significant. The same trend was also observed, slightly increase or no change, with the other markers signalizing an immature state of the cells, i.e., *ZFP521* and *SOX9* (exception in 8B with a decrease). In parallel, the relative expression level of *PPARG*, the master regulator of adipogenesis, slightly decreased in all 3D conditions tested. The other prodifferentiation markers also followed this trend, i.e., *ZFP423* and *DKK1*, as shown in Figure 9A,C. Instead, for the cells cultured in 3D on PNF MC, these two genes’ relative expression levels slightly increase (Figure 9B). Finally, the value of *RUNX2* slightly decreased or remained unchanged, indicating that the hASCs grown in 3D do not randomly differentiate into chondrocytes or osteocytes. Therefore, since no statistically significant differences between genes involved in hASCs differentiation were observed, we concluded that the undifferentiated status of hASCs is preserved very well when they grow on MCs in defined serum-free conditions. However, this profile worsens with increasing cell density [23]. Therefore, an important goal for the future will be to learn how to culture hASC on MC and maintain them at the ideal density to keep their undifferentiated status until harvest or other downstream processes. In conclusion, hASCs grew very well on the biodegradable *BR44* MC in a static or dynamic cell culture system combined with the defined *UrSuppe* medium.

### 3.6. Investigating the Secretome Profile of hASCs Cultured in Standard Static 2D vs. Static or Dynamic 3D Conditions

**Figure 10:**
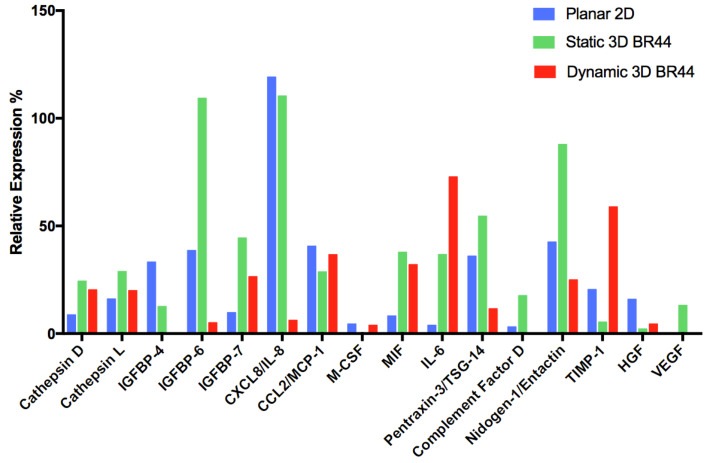
Representative quantitative secretome analysis of hASCs cultured in defined xeno- and serum-free conditions (*UrSuppe* medium). Human ASCs’ supernatants of cells at passage 2 were analyzed using a commercially available “Proteome Profiler Human Adipokine Array.” The three cell culture conditions tested were standard 2D cell culture system, blue bars; static 3D conditions on *BR44* MC, green bars; dynamic 3D conditions on *BR44* MC, red bars. Data are represented as relative Mean Pixel Intensity (MPI) of fluorescence. Background fluorescence was subtracted from each measured value and then normalized by the positive control intensity to obtain the relative MPI. This experiment was performed with hASCs obtained from the biopsy of one donor. More information about these adipokines could be found in Supplementary Materials Tables S9 and S10.

To determine the presence of 58 human adipokines, we employed a commercially available “Proteome Profiler Human Adipokine Array” kit (see Table 2, Figure 10, and Supplementary Materials Figure S8). Thus, we decided to challenge the results obtained previously by flow cytometry and RT-qPCR by analyzing the hASCs cultured under defined conditions on MC with a completely different technique, i.e., the study of the polypeptides secreted by these cells. Interpreting and making biological sense for a proteome profiler array is very complicated, especially when many secreted factors are detected. Fortunately, our case was not too complex and allowed us to notice patterns that could help interpret the results. Thus, we realized that the detected factors could be classified into six categories. Furthermore, they were all polypeptides associated with the adipose tissue (for review, see [43]). As a first very positive result, we found that two signature adipokines (adiponectin and leptin, reviewed in [44]), typical for mature cells, were not detected. Moreover, the signal corresponding to Nidogen/Entactin, a sulfated multidomain glycoprotein component of basement membranes, was reduced and feeble when the cells were grown in dynamic 3D conditions.

In this assay, we noticed that IGFBP-4 is not present in the supernatant of hASCs cultured in dynamic 3D conditions, and besides, IGFBP-6 is reduced compared to the other two conditions. On the other hand, IGFBP-7 secretion was higher when the hASCs were cultured on *BR44* MC, whereby static 3D was better than dynamic 3D. Compared to the standard 2D cell culture system, we consider its upregulation in both 3D conditions as an interesting and positive phenomenon.

The protein microarray used in this study evidenced the presence of several cytokines [45] and chemokines [46] in the tested media: CXCL8/IL-8, CCL2/MCP-1, M-CSF, MIF, IL-6, PTX3/TSG-14, and Complement Factor D. It is challenging to find a common denominator for these secreted factors, which are primarily associated with an inflammatory condition. However, we noted some differences depending on the cell culture conditions. The expression of IL-6 is low in the conditioned media of cells grown in 2D, whereas higher in those from the 3D conditions (dynamic > static). IL-6 is a pleiotropic cytokine and takes on essential tasks in immunity, tissue regeneration, and metabolism [47]. Like several other inflammatory cytokines, it is an inhibitor of adipogenesis and blocks adipocyte differentiation [48]. Recently, it was shown that IL-6 mediates white adipose tissue browning [49]. Therefore, this cytokine plays an important regulatory role in adipose tissue, and much of these effects have yet to be understood. Since IL-6 is an antiadipogenic factor and plays a role in angiogenesis [21], the increase in its secretion under dynamic 3D conditions could be considered positive as it would help avoid unwanted spontaneous differentiation into adipocytes of cultured hASCs. Regarding IL-8, it is high in 2D and static 3D conditions, whereas very low in the dynamic 3D cell culture system. Pentraxin 3 (PTX3), also called tumor necrosis factor (TNF)-inducible gene 14 protein (TSG-14), is a pattern recognition molecule that acts in innate immunity and is a valuable biomarker of a proinflammatory status [50]. This factor was promptly found in the supernatants of cells cultured in 2D and static 3D, but was lower in dynamic 3D conditions. The adipose tissue produces complement components that play nonimmunogenic roles [51]. Indeed, it was shown that Complement Factor D promoted adipocyte differentiation [52]. In our analysis Complement Factor D was expressed only in static 3D conditions. In 2D it was very low, whereas, in dynamic 3D conditions, it was not detected. Finally, MCP-1 was expressed similarly in all conditions tested as well as M-CSF, albeit at moderate levels.

In our study, TIMP-1 expression is higher in the supernatants of hASCs grown on *BR44* MC under dynamic 3D conditions than standard 2D culture systems and 3D static conditions. We evaluated this result positively, marking one more point in favor of the *BR44* MC and the defined dynamic 3D cell culture system. Finally, in the cell culture supernatants, we also detected two factors related to angiogenesis and cell proliferation: Vascular Endothelial Growth Factor (VEGF, reviewed in [53]) and Hepatocyte Growth Factor (HGF, reviewed in [54]). The first one was detected only in the conditioned media of hASCs cultured in static 3D conditions. In contrast, the second one was present in the supernatants of hASCs grown in standard 2D cell culture vessels but very low in both 3D cell culture conditions. Table 2 qualitatively displays the results obtained for each detected factor and catalogs them according to six biological characteristics to facilitate the comparison of the three secretion profiles. A careful analysis of this table’s trends suggests that antiadipogenic factors increase while adipogenic and inflammatory factors decrease in dynamic 3D conditions. These observations are in line with the results obtained by RT-qPCR and flow cytometry.

### 3.7. Comparing the Amino Acid Consumption between hASCs Cultured in a Standard 2D System with Those Grown in Dynamic 3D Conditions on BR44 MC

**Figure 11:**
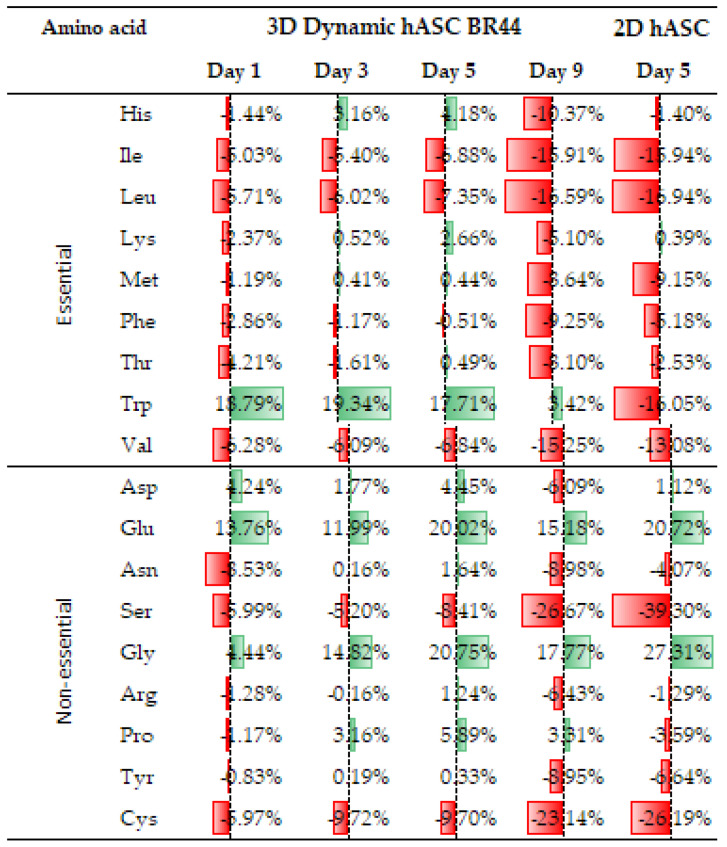
Amino acid profile overview. The values were calculated relative to the initial amino acid concentrations in the *UrSuppe* medium. Red: consumed amino acids; Green: produced amino acids. Time-dependent profiles of each amino acid are shown in Supplementary Materials Figure S9.

We reasoned that free AAs in hASCs culture media could provide valuable information on cell metabolism and obtain information on their status by comparing the AAs consumption profile obtained with cells grown in static 2D with cells cultured in dynamic 3D conditions (see Figure 11). Furthermore, this analysis may reveal limiting nutrients that may inhibit cell growth, thus indicating how to improve the cell culture medium’s performance. As shown in Figure 11, the amino acid consumption of hASCs grown in dynamic 3D conditions was monitored with samples taken on days 1, 3, 5, and 9. Instead, the consumption in standard 2D conditions was determined on day 5. As a first observation, we noticed that amino acids’ trends and consumption patterns are similar between the standard 2D system and the dynamic 3D conditions. We consider this an excellent result, which indicates, once more, that hASCs grown in dynamic 3D conditions do not degenerate and maintain characteristics similar to those of cells cultured with traditional 2D static systems.

The amino acid consumption profile in Figure 11 shows that the essential AAs are generally consumed during the cell cultivation period, in both planar 2D and dynamic 3D conditions in a similar way. Some of the nonessential amino acids are, in reality, essential for synthesizing nucleotides (glycine, aspartate, and glutamine). However, their concentration does not matter as it remains constantly higher than the initial one or drops slightly at the end of the cultivation period (Asp, day 9, dynamic 3D). Certain amino acids often accumulate in the medium during batch growth with surplus glucose and glutamine [55]. This may be due to a strong overflow metabolism originating from high concentrations of AAs. Indeed, the Ala-Gln dipeptide (“Stable Glutamin” at the concentration of 200 mM) added to our medium was split entirely very quickly into Ala and Gln; this led to a rapid, significant increase of Ala and Gln concentration in the medium (see Supplementary Materials Figure S9). Consequently, there is a large excess of these two molecules in the cell culture medium, and hence, they are not considered further in this analysis. On the other hand, glutamate and glycine are secreted in the cell culture media regardless of the culture system used to grow the hASCs. The amino acid consumption profile of hASCs cultured in dynamic 3D conditions is similar to that obtained from traditional planar 2D cultures, and the mainly consumed are Ser and Cys. Our data are qualitatively in good agreement with measurements of Schop et al. [56] and Higuera et al. [57]. None of the AAs are exhausted, and the lowest concentration, that of serine, still represents around 70% (for dynamic 3D) or 60% (for planar 2D) of the initial concentration. Accordingly, growth inhibition by an amino acid is unlikely. Therefore, this analysis suggests that the *UrSuppe* medium has a composition of amino acids sufficient to sustain the growth of hASCs. The only surprising observation is represented by the tryptophan level, which is higher in dynamic 3D set up than the planar 2D condition. We have no explanation for this phenomenon, and we can only take note of it.

## 4. Discussion

Regenerative medicine needs new tools to obtain high cell numbers while preserving the behavior of the cell. It is well known that intrinsic molecular mechanisms regulate the fate of stem cells. However, many cell-external factors are also responsible for the overall orchestration of stem cell activity. Especially, the ECM affects stem cell fate, with specific relevance on ECM ligands’ interactions with cell surface receptors [56]. This ECM-based control of the cell may also happen through various physical mechanisms: ECM geometry, ECM elasticity, and mechanical signals conveyed from the ECM to the cell. Furthermore, cells can sense micro- and nano-scale geometric stimuli from their surroundings, which may occur in the form of differences in molecular conformation, for example, such as surface topography, surface roughness, and fiber diameter [58]. The stiffness of the ECM may also participate in lineage commitment, and this hypothesis was tested recently. Interestingly, it turned out that a soft substrate reproducing the tissue niche could keep stem cells in a quiescent status while safeguarding their “stemness” [58].

This study used the commercial non-biodegradable PNF MC as a “gold standard” and positive control to evaluate the experiments’ outcomes. The *BR44* and PNF MCs’ stiffness and permeability are very different: PNF offers the cells a rigid and impermeable adhesion surface, while *BR44* is softer and more permeable since it is very porous. Thus, these two MCs’ differences are attributable to their composition: resorbable biomaterial of natural derivation (*BR44*) versus nondegradable synthetic material (PNF). MCs’ structure and composition are essential as the adhesion substrate is one of the primary influencers of cell expansion and differentiation. Furthermore, it is known that the stiffness of the substrate where the cells grow can influence their cell differentiation [58,59]. Since the adipose tissue can be considered as a soft environment, it may be possible that a soft adhesion surface would favor hASCs growth. Therefore, it could be possible that a MC based on natural biopolymers mimics the ECM properties (a reticulated porous shape and a soft structure, like *BR44*) better, and hASCs benefit from this environment.

Due to safety concerns and possible uncontrollable detrimental effects of FBS in clinical applications [60,61,62], the *BR44* MC was used in combination with a proprietary defined xeno- and serum-free cell culture medium (*UrSuppe*) suitable for the dynamic cultivation of hASCs in spinner flasks. We believe that developing the MC and the defined cell culture medium simultaneously is an excellent strategy. Indeed, it has been recently shown that a good performing serum-free medium is cell-specific and demands optimization for MC-based dynamic cell culture systems [18]. The tissue cells, under normal conditions, are never in direct contact with the serum. The only ones that are constantly so are blood and endothelium cells. Therefore, the tissue cells are surrounded by the interstitial fluid, which contains fewer proteins than serum and is specific for every tissue or organ. This is because each interstitial fluid is formed by the tissue’s secretion products with which it is in contact and therefore acquires a particular blend of secreted proteins and metabolic molecules [63,64]. Therefore, a serum-free medium tries to mimic and reproduce an interstitial fluid specific for a particular type of tissue cell.

Cells are usually organized in complex 3D structures and are subjected to movements: fluids flow between the cells, tissue contractions or movements are not uncommon, and motor activities are widespread actions in the animal kingdom. Thus, it may be possible that the conditions created in a stirred flask or agitated bioreactor somehow better reproduce the natural situation familiar for the cells in the body. Therefore, a dynamic 3D cell culture system combined with a defined cell culture media may mimic better the natural cells’ environment allowing hASCs to proliferate and maintain their undifferentiated status.

This study was also instrumental in understanding the drawbacks of biodegradable microcarriers compared to traditional ones that are not bioerodible. Indeed, we noticed that *BR44* MC is more challenging to handle than its synthetic counterparts. The biodegradable MC easily sticks to pipettes and other equipment commonly used in the laboratory. However, this peculiarity also characterizes other MCs made from organic or biological polymers, such as Cultisphere S and G, Cytodex 3, and Celnest Macroporous MCs (Fujifilm).

This intrinsic predisposition to stick together is also maintained in culture with cells by this type of MCs. Thus, even under dynamic conditions, this can result in the formation of large aggregates. Therefore, it is necessary to fine-tune the stirring conditions to minimalize this phenomenon, which could cause a decrease in cell density due to limiting diffusion of nutrients to the center of large cell-MC clusters. Therefore, MCs manufactured with a biopolymer may need a more laborious setup regarding the seeding densities and agitation parameters than classic synthetic MCs. This observation suggests that perhaps stirred tank bioreactor may not be the most suitable system for growing hASCs when biodegradable MCs are used. Therefore, it will be interesting to test other types of reactors that may better adapt to biodegradable MCs’ peculiarities [65]. As an alternative, there would be the possibility of culturing hASCs without scaffolds, in suspension, in the form of cell aggregates or spheroids [66,67]. This cell culture technique is starting to give encouraging results, but it is unlikely that it will work for all types of primary (stem) cells. Moreover, it will be challenging to reach such high expansion factors with this technique, as in cultures based on MC.

A comparison of the obtained growth-related parameters of this proof-of-concept study with literature data clearly showed the suitability of the *BR44* MC for the in vitro expansion of hASCs (see Table S11 in the Supplementary Materials). As for different non-biodegradable MCs in serum-free media, comparable data were achieved at a small scale [21,23,68,69]. However, it is clear that further investigations, especially in larger bioreactor systems, are required to improve the process performance with *BR44* MC further. This is particularly important in optimizing the MC suspension properties (i.e., MC concentration, cell-to-bead ratio) to increase the total cell yield to achieve clinically relevant cell numbers in fully automated and instrumented bioreactor systems.

The growth rate is not the only parameter that is important in the cell culture process. A crucial feature for a stem cell culture platform is its ability to “seal” the cells’ undifferentiated status during the in vitro amplification phase. Therefore, it was imperative to verify by flow cytometry whether the hASCs growing on *BR44* MC expressed the same standard surface markers and maintained an immature status as the same batch of cells expanded in traditional cell culture vessels (2D conditions). Interestingly, this analysis highlighted some notable deviations between cells grown in 2D from those grown in 3D on *BR44* MC or PNF MC in static conditions. First, CD105 expression was significantly reduced when hASCs were grown on PNF MC. We believe that this effect is mediated by the composition of the different materials that form the two different MCs. However, the downregulation of CD105 in hASCs grown on PNF MCs does not automatically mean that the cells are no longer usable for cell therapies, as reported by Liem Hieu Pham et al. [70]. Secondly, the reduction of CD146 expression on cells grown on MC was intriguing. This surface protein is also referred to as melanoma cell adhesion molecule (MCAM). CD146 occurs with two transmembrane isoforms and a soluble protein detectable in the plasma. The traditional role attributed to it is to participate in intracellular adhesion. However, CD146 mediates pleiotropic tasks through homophilic and heterophilic interactions with polypeptides present in the surroundings [71,72]. Several reports suggested that CD146 may be linked with many nonresolving inflammatory diseases [73,74]. It was also described on the surface of activated inflammatory cells [75,76]. Yongting Luo et al. found that macrophagic CD146 promotes foam cell formation and interacts with CD36 to mediate oxidized low-density lipoprotein (oxLDL) uptake [77]. Consequently, it is not surprising that CD146 was also associated with adipogenic differentiation. Indeed, Graham G. Walmsley et al. showed that hASCs induced to differentiate into adipocytes expressed the CD146 as well as the CD36 marker [24]. This latter also marks adipocyte progenitors with pronounced adipogenic potential and triglycerides storage ability and plays a functional role in adipogenesis [27,28,78]. Indeed, CD36 expression was upregulated when hASCs start to differentiate into adipocytes [24], and it was strongly expressed by mature adipocytes [26]. Its expression is also upregulated in a wide range of cell and tissue types by various senescent stimuli [79]. For these reasons, we consider the presence of CD36 on hASCs as a label of loss of the undifferentiated status and ongoing adipogenesis. Finally, we confirmed that CD36 and CD146 could be coexpressed on early adipocytes, as shown in the Supplementary Materials Figure S5. The low expression of CD15 or CD34 observed is also an excellent feature of the hASCs grown in *UrSuppe* medium, which signalize being mostly immature cells. CD15, also known as Sialyl LewisX (sLeX) or stage-specific embryonic antigen 1 (SSEA-1), is a tetrasaccharide carbohydrate commonly linked to O-glycans on the surface of cells. Experiments gave this surface marker a role in cell adhesion and the regulation of cell differentiation [80]. Remarkably, its expression pattern is different from humans and mice. Indeed, in mice, it is present on embryonic stem cells (ES), embryonal carcinoma cells (EC), 8-cell to blastocyst embryos, a subset of embryonic inner cell mass, and it disappears upon differentiation. However, in humans, CD15 is not detected on undifferentiated ES cells, but its transcription is upregulated along with differentiation [81,82]. CD15 is highly expressed in adult human granulocytes. The transmembrane phosphoglycoprotein CD34 was first characterized as a hematopoietic stem cell marker but was later found on several nonhematopoietic cell types [83]. This marker is associated with selecting and enriching stem cells for bone marrow transplants in the clinic. Freshly extracted hASCs are CD34^+^, but they lose this marker upon plastic adhesion and culture [84]. In the adipose tissue, CD34 is expressed on the surface of mature adipocytes [26], and it is upregulated when hASCs are induced to mature toward an adipogenic lineage [24,85]. These data suggest that CD34, at least in the adipose tissue, does not signal “stemness” but rather a marker of adipogenic differentiation expressed by early and mature adipocytes.

Similar to flow cytometry, we used RT-qPCR to monitor stemness or differentiation-related genes. The hASCs used during this project were obtained from subcutaneous adipose tissue. Thus, we expected that the default differentiation pathway of these cells, in case of spontaneous maturation in vitro, was towards the tissue from which they were purified. Adipogenesis is a complex and multistage process through which precursor cells differentiate into mature adipocytes, coordinated by signaling interchanges among a myriad of different complex molecular systems [41]. Some of the genes that orchestrate this process by inducing or repressing adipogenesis are known and are schematically represented in Figure S7 and Tables S7 and S8 in the Supplementary Materials. Even with these experiments, we found that 3D conditions did not compromise the cells’ undifferentiated status. On the contrary, in several respects, it appeared even superior to that of hASCs grown in standard 2D cell culture vessels. These experiments proved again that the substrate on which the cells were cultured could modulate some genes’ expression, as observed with the flow cytometry analysis.

Since some polypeptides are secreted only at particular stages of development or maturation, they may be of diagnostic value in revealing a specific metabolic state or a specific degree of cell differentiation. One of the earliest changes seen in adipocyte differentiation is the biogenesis of the basement membrane. Nidogen/Entactin is an excellent marker to signalize ongoing adipogenesis [86]. Therefore, this protein’s low expression level, together with the lack of adiponectin and leptin, proves that most cells stayed undifferentiated and immature in the dynamic 3D cell culture system. Next, we noticed the presence of two Cathepsin family members. These polypeptides are proteases that are involved in crucial mammalian cellular turnover. Cathepsin D [87] (aspartyl protease) and Cathepsin L [88] (cysteine protease) play essential physiological roles in the adipose tissue and are essential for remodeling the extracellular matrix (ECM) [89] during adipogenesis. Interestingly, Cathepsin D also has mitogenic activity independent of its proteolytic activity, and its knock-out in mice revealed that it is indispensable for postnatal tissue homeostasis [90]. Therefore, the presence of these proteins in the supernatants is not surprising, and there are no relevant differences between the three cell culture systems considered in our study. However, these proteases are considered to be promoters of adipogenesis [87,88]. The next group of related polypeptides consists of the Insulin-like growth factor-binding proteins (IGFBPs). One of their primary function is to fine-tune Insulin-like growth factors (IGF-I and IGF-II) action by regulating the accessibility of these peptide hormones to their receptors [91]. Both IGF-I [92] and IGF-II [93,94] are proadipogenic. It has been reported that IGFBP-4 interacts with IGF-I and its presence negatively correlates with adipose tissue growth [95]. IGFBP-6 can bind IGF-II and IGFBP-7 both, IGF-I and II [96]. Thus, it is likely that these three members of the IGFBP family are antiadipogenic. However, IGFBP-6 secretion decreased in the dynamic 3D conditions with *BR44* MC, and this could be an unfavorable trend. Nevertheless, since it does not serve to sequester IGF-II as this proadipogenic growth factor is not present in the *UrSuppe* medium, its decrease should not affect the undifferentiated status of the hASCs. Furthermore, another member of the family, IGFBP-7, remains well expressed in the dynamic 3D conditions and may compensate for the decrease of IGFBP-6. IGFBP-7, also called Angiomodulin (AGM), is a particular member of this family. It is a secreted polypeptide kept in Weibel-Palade granules and produced by the developing vasculature [97]. It can also bind chemokines and growth factors, including VEGF-A [98,99]. Therefore, IGFBP-7 is now considered proangiogenic in the developing vasculature, that in combination with VEGF-A and presumably other angiogenic factors, it guides and stabilizes the nascent vasculature. The data collected so far support a model wherein IGPBP-7 may serve as biochemical growth factors storage by providing an extracellular scaffold to which VEGF-A and other proteins could be deposited within the ECM milieu, resulting in the proper downstream signaling and fine-tuning of critical biological processes, such as angiogenesis [100]. Taken together, the secretion profile of IGFBPs family members appears to be favorable to maintaining “juvenile” hASCs. Macrophage migration inhibitory factor (MIF) is a multifunctional cytokine that controls proinflammatory response and is linked with numerous inflammatory and autoimmune diseases [101]. During adipogenesis, the role of MIF was studied by Atsumi et al. and found to be antiadipogenic [102]. This finding agrees with the known ability of MIF to counter-regulate the immunosuppressive effects of glucocorticoids [103], which are also well known for their capacity to induce adipocyte maturation. Interestingly, this factor’s expression was higher in the samples where the cells were grown in both 3D conditions. ECM remodeling marks out the onset of the differentiation during adipogenesis: type I and III collagen, fibronectin, and β1-integrins are downregulated, whereas type IV collagen and nidogen-1/entactin are upregulated. This guided turnover of the ECM is orchestrated by matrix metalloproteases (MMP) and by tissue inhibitors of metalloproteases (TIMP), which control the proteases’ enzymatic activity [86]. TIMP-1 was the first described natural collagenase inhibitor. However, later it was shown that TIMP-1 is a negative regulator of adipogenesis [104,105]. Surprisingly, it was also discovered that TIMPs function also as signaling molecules with cytokine-like activities, thus playing important roles in various biological processes, such as cell growth, apoptosis, differentiation, angiogenesis, and oncogenesis. All these functions are independent of MMP inhibition [106,107]. Therefore, TIMP-1 is a molecule with a biological significance not to be underestimated and not just a protease inhibitor. From our perspective, if the secretion of an antiadipogenic factor increases in 3D dynamic conditions compared to the static 2D or 3D system, we consider it a positive event since it could be helpful to keep hASCs undifferentiated. The increase of TIMP-1 in the dynamic 3D condition is therefore considered positively. In summary, these results indicate that the secretion profile can be modulated depending on the cultivation system (standard static 2D vs. static 3D vs. dynamic 3D). The commercially available “Proteome Profiler Human Adipokine Array” we used for our study detects up to 58 adipose tissue-related molecules; however, we only found 16. We think that this result may denote that the cells cultured with the three different systems remain immature, and their relatively simple secretome manifests this undifferentiated status. We wanted to verify, with a technique different from flow cytometry or RT-qPCR, whether hASCs grown in dynamic 3D conditions maintained their undifferentiated status at least as much as those grown in a standard 2D cell culture system. This seems the case since the secretion profiles are similar.

In recent years, it has been realized that many nutrients and intermediates of energy metabolism are not only carriers of energy. They also function as molecular mediators by activating specific receptors that fine-tune metabolic pathways, immune and cellular signaling, and cell fate’s epigenetic regulation [108,109]. Different cell states need specific metabolic programs to sustain the distinct bioenergetic demands underlying their specialized functions. Amino acids (AAs) are also essential as regulators of fluxes through major metabolic pathways [110,111]. The amino acid consumption profiles we recorded by analyzing the supernatants fell qualitatively in with measurements of Schop et al. [56] and Higuera et al. [57]. However, there was one intriguing surprise: The tryptophan concentration was higher in dynamic 3D set up than in planar 2D condition. A recent study has shown that the tryptophan metabolism in mesenchymal stem cells modulates inflammation by suppressing leukocytes’ activation [112,113]. It could be possible that the dynamic 3D arrangement promotes the secretion of this amino acid that then contributes to the anti-inflammatory effect seen in previous experiments. Interestingly, Higuera et al. [57] observed a similar phenomenon using bone marrow-derived MSCs and comparing amino acid consumption between static and dynamic cell culture systems. Indeed, it was found that cysteine and proline are produced by bone marrow-derived MSCs in static while consumed under dynamic culture conditions. These data suggest that the static vs. dynamic mechanical treatment of cells grown with the same medium and conditions can also influence some amino acid consumption or production.

Cell culture is a young science that has its origins in pioneering studies that began around 120 years ago [114]. Even today, it still relies on the use of indefinite culture media containing FBS and 2D static conditions based on polystyrene cell culture vessels. However, about 40 years ago, the need to have cells that produce large quantities of therapeutic biomolecules made it necessary to introduce new techniques, such as defined xeno- and serum-free media, microcarriers, and different types of bioreactors to culture them. Recently, the idea of using cells for regenerative and cosmetic medicine, or cellular agriculture, has enormously boosted the interest in these technologies. Unfortunately, our knowledge of these new processes is still limited. Indeed, we still rely heavily on procedures and knowledge related to traditional static 2D techniques combined with undefined FBS-based culture media. Therefore, our primary concern in this feasibility study was to verify whether primary hASCs grew on *BR44* MC under defined dynamic conditions, maintaining features close to those grown in static 2D cell culture vessels, which is the system we know best and is the primary reference for all laboratories worldwide. We tested more than 50 microcarrier prototypes over 2 years before deciding to complete the study with the *BR44* MC. Simultaneously, we developed the cell culture medium we called *UrSuppe*.

In conclusion, our data suggest that hASCs cultured on *BR44* MCs in xeno- and serum-free medium under dynamic conditions do not degenerate and maintain similar characteristics to those grown in traditional static 2D systems. Surprisingly, hASCs preserved their undifferentiated status better regarding some molecular markers and were less inflammation-prone in dynamic 3D conditions than in the static 2D setup. This means that the most advanced dynamic 3D cell culture technologies can be employed to mass expand hASCs for future cell therapies. Furthermore, the *UrSuppe* medium could prove very useful for studying ex vivo human adipocytes’ biology and their precursors under defined cell culture conditions.

## Figures and Tables

**Table 1:** Growth parameters of *BR44* in Spinner flask culture.

Parameter	*μ*	*t_d_*	*Att_eff_*	*X_max_*	*EF*	*Y_Lac/Glc_*	*q_Glc_*	*q_Lac_*	*q_Amn_*
	(d^−1^)	(d)	(%)	(10^5^ cells/mL)	(-)	(mmol/mmol)	(pmol/cell/d)	(pmol/cell/d)	(pmol/cell/d)
*BR44*	0.25	2.8	188	3.18	8.1	2.6	3.60	10.01	1.87

*µ*: specific cell growth rate. *t_d_*: doubling time of cell population. *Att_eff_*: attachment efficiency. *X_max_*: maximum cell concentration on the planar growth surface. *EF*: expansion factor. *Y_Lac/Glc_*: lactate yield per glucose equivalent. *qGlc*: specific glucose consumption rate. *qLac*: specific lactate production rate (growth-dependent). *qAmn*: specific ammonium production rate (growth-dependent).

**Table 2:** Simple qualitative evaluation of the factors secreted by hASCs grown in three systems: standard 2D cell culture system, static, or dynamic 3D conditions. The qualitative evaluation for each detected factor is indicated by the number of X. Shallow expression is indicated by (X), whereas no expression by (-). The *UrSuppe* medium was employed in all conditions tested. Quantitative results are shown in Figure 10. For more details, see Supplementary Materials Figure S8 and Tables S9 and S10.

Secreted Protein Type	Analyte/Control	2D hASC	3D Static hASC *BR44*	3D Dynamic hASC *BR44*
Aspartyl- and cysteine-proteases	Cathepsin D	X	XX	XX
	Cathepsin L	X	XX	X
Insulin-like growth factor binding proteins	IGFBP-4	XX	X	(-)
	IGFBP-6	X	XXX	(X)
	IGFBP-7	X	XXX	XX
Cytokines, chemokines, inflammatory factors	CXCL8/IL-8	XXX	XXX	(X)
	CCL2/MCP-1	X	X	X
	M-CSF	X	(-)	X
	MIF	(X)	XXX	XXX
	IL-6	(X)	XX	XXX
	Pentraxin-3/TSG-14	XX	XXX	X
	Complement Factor D	X	XXX	(-)
Extracellular matrix	Nidogen-1/Entactin	XX	XXX	X
Metalloproteases inhibitor	TIMP-1	XX	X	XXX
Angiogenesis and cell proliferation	HGF	XX	(X)	X
	VEGF	(-)	X	(-)

## Data Availability

The data presented in this study are available on request from the corresponding author.

## Supplementary Materials

The following are available online at https://www.mdpi.com/article/10.3390/jfb12020025/s1, Figure S1: A pluriStrainer Mini 40 µm is used to separate MCs from the nuclei; Figure S2: Flow cytometry analysis of CD36 and CD146 in hASCs; Figures S3–S5: Single-parameter histogram for flow cytometry analysis of expanded hASCs; Figure S6: Average flow cytometry expression; Figure S7: Schematic illustration of adipogenesis; Figure S8: Comparing the adipokines secretion profile of hASCs; Figure S9: Time-dependent profiles of the amino acid concentration during the cultivation under dynamic and planar conditions. Table S1: Detailed information of the antibodies used for the flow cytometry measurements; Table S2: Reverse transcription detailed procedure; Table S3: Primer sequences; Table S4: RT-qPCR cycle conditions; Table S5: Materials; Table S6: Percentages of nuclei found in G1, G2, or in S phase; Table S7: Overview of measured stemness maintenance genes; Table S8: Overview of measured differentiation regulators/markers; Table S9: Overview of secreted adipokines and chemokines; Table S10: Human adipokine array; Table S11. Overview of cell culture results achieved in MC-based expansion processes with serum-free cell culture media.
