# Supplementary material for: Human Adipose Stem Cells (hASCs) Grown on Biodegradable Microcarriers in Serum- and Xeno-Free Medium Preserve Their Undifferentiated Status
Source: J Funct Biomater. 2021 Apr 16;12(2):25. doi: 10.3390/jfb12020025 (PMC8167760; doi:10.3390/jfb12020025)
Supplement: Supplementary file 1 [file jfb-12-00025-s001.zip › jfb-1107682-SI.pdf]

Journal of
Functional Biomaterials

MDPI

Supplementary Materials for the Materials and Methods Section

Table S1. Detailed information of the antibodies used for the flow cytometry measurements

Name	# Catalog	Company	Concentration for SVF [ng]	Concentration for ASC [ng]
7-AAD	559925	Becton Dickinson	2.5 µL	2.5 µL
CD15-FITC	332778	Becton Dickinson	-	50
CD34-BV650	343624	BioLegend	125	-
CD34-PE	130-081-002	Miltenyi	-	50
CD36-APC	130-095-475	Miltenyi	55	50
CD45-PC7	304016	BioLegend	125	-
CD61-PE	IM3605	Beckman & Coulter	-	50
CD73-FITC	344016	BioLegend	75	50
CD90-APC	328113	BioLegend	-	50
CD105-PE	323206	BioLegend	-	50
CD146-PE	130-092-853	Miltenyi	34	50
SYTO™40	S11351	Thermo Fisher	5 µM	-

Evaluation of cell proliferation

It is often challenging to detach cells efficiently from the MCs on which they grew. For this reason, we developed a protocol based on nuclei count: A cell lysis buffer solution frees the nuclei that can thus be enumerated by flow cytometry. This method also allows assessing the cell cycle status of the collected nuclei. So, it is possible to determine the proliferative status of the cells grown on the MCs.

Protocol: Nuclei count after cell lysis.

1. Collect the MCs + cells from each well and transfer them in 1.5 mL Eppendorf tubes. Rinse each well twice with PBS to recover all the MCs + cells.
2. Centrifuge at 400 g for 5 min at RT.
3. Discard the supernatant and resuspend the pellet (microcarriers + cells) in 1 mL of "Lysis & Nuclei Extraction Buffer" (LNEB; 0,2 M Citric acid + 2% Triton X-100).
4. Incubate 5-10 min at RT and pipette up and down until the cells are completely lysed.
5. Add the lysate to a pluriStrainer Mini 40 µm inserted into a 5 mL Eppendorf tube (pluriSelect, cat. no. 43-10040-60, see figure S1). The strainer separates microcarriers from cell lysate/nuclei. Wash filters twice with LNEB to recover all the nuclei.
6. Centrifuge at 800 g for 5 min at RT (5 mL Eppendorf + filter) to collect the nuclei.
7. Resuspend the pellet (nuclei) in 100 µL of CLNEB.
8. Stain with 7-AAD for 5 min at RT, and analyze by flow cytometry. Nuclei analysis: discrimination between G1, G2, and S phases.

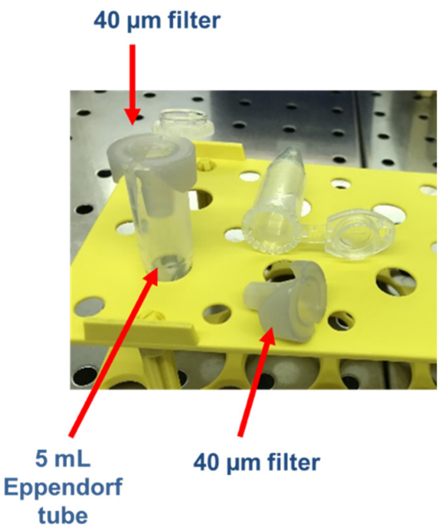

Figure S1. A pluriStrainer Mini 40 µm is used to separates MCs from the nuclei. The centrifugal force helps to collect the nuclei quantitatively on the bottom of a 5 mL Eppendorf tube.

Table S2. Reverse transcription detailed procedure

Mix 1	
**Reagent**	**Amount**
RNA	Up to 5 µg
Oligo dT	0.5 µg
Random Primers	0.5 µg
H_2O	Final volume 5 µL

Mix 2	
**Reagent**	**Volume**
Buffer 5x	2 µL
$MgCl_2$	1 µL [2.5 mM]
dNTPs	0.5 µL [0.5 mM]
Inhibitor RNasi	0.25 µL [20 Units]
RT Enzyme	0.5 µL
H_2O	Final volume 5 µL

Procedure:

Add Mix 1, incubate 5' at 70 °C, cool to 10 °C and incubate 5' in ice.

Add Mix 2 and incubate 5' at 25 °C, 42 °C for 1h, and 70 °C for 15'.

Table S3. Primer sequences

**Gene Name**	**Forward Primer (5′-3')**	**Reverse Primer (5′-3')**
ACTB	CTG GAA CGG TGA AGG TGA CA	AAG GGA CTT CCT GTA ACA ATG CA
SOX9	AGC GAA CGC ACA TCA AGA C	CTG TAG GCG ATC TGT TGG GG
RUNX2	TCA ACG ATC TGA GAT TTG TGG G	GGG GAG GAT TTG TGA AGA CGG
PPARG	GCT TTC TGG GTG GAC TCA AGT	GAG GGC AAT CCG TCT TCA TCC
PREF1	TGA CCA GTG CGT GAC CTC T	GGC AGT CCT TTC CCG AGT A
ZFP423	GAT CAC TGT CAG CAG GAC TT	TGC CTC TTC AAG TAG CTC A
ZFP521	GGC TGT TCA AAC ACA AGC G	GCA CAT TTA TAT GGC TTG TTG
DKK1	ATA GCA CCT TGG ATG GGT ATTC C	CTG ATGA CCG GAG ACA AAC AG

Table S4. RT-qPCR cycle conditions

Phase	T (°C)	Time (min)	Repetition
Denaturation	95 °C	2:00	
Denaturation	95 °C	0:05	X 40
Annealing+ Extension	60 °C	0:20	
Denaturation	95 °C	0:05	
Melting Curve	65-95 °C	18:00	

Evaluation of growth-related parameters

Equation used to calculate the growth-related parameters:

(I) Specific growth rate μ (Eq. 1)

$$\mu = \frac{ln\big(X_A(t)\big) - ln\big(X_A(0)\big)}{\Delta t} \quad (1)$$

Where μ is the net specific growth rate. XA(t) and XA(0) are the cell numbers at the end and the beginning of the exponential growth phase, respectively, and t is the time.

(II) Doubling time t_d (Eq. 2)

$$t_d = \frac{ln(2)}{\mu} \quad (2)$$

Where *td* is the doubling time, ln(2) the binary logarithm of 2, and μ the specific growth rate.

(III) Population Doubling Level PDL (Eq. 3)

$$PDL = \frac{1}{log(2)} \cdot log\left(\frac{X_A(t)}{X_A(0)}\right) \quad (3)$$

PDL is the number of population doublings, and XA(0) and XA(t) are the cell numbers at the beginning and end of the cultivation.

(IV) Expansion factor EF (Eq. 4)

$$EF = \frac{X_A(t_{max})}{X_A(t=0)} \quad (4)$$

EF is the expansion factor, and $X_A(t_{max})$ is the maximum cell number, and $X_A(t=0)$ is the inoculated cell number.

(V) Lactate yield from glucose $Y_{Lac/Glc}$ (Eq. 5)

$$Y_{Lac/Glc} = \frac{\Delta Lac}{\Delta Glc} \quad (5)$$

Where $Y_{Lac/Glc}$ is the lactate yield from glucose, ΔLac is the lactate production over a specific time period, and ΔGlc is the glucose consumption over the same time period (= exponential growth phase)

(VI) Specific metabolite flux q_{met} (Eq. 6)

$$q_{met} = \left(\frac{\mu}{X_A(t)}\right)\left(\frac{C_{met}(t) - C_{met}(0)}{e^{\mu t} - 1}\right) \quad (6)$$

Where q_{met} is the net specific metabolite consumption or production rate (for Glc, Lac, Amn), μ is the specific cell growth rate, XA(t) is the cell number at the end of the exponential growth phase; $C_{met(t)}$ and $C_{met(0)}$ are the metabolite concentrations at the end and the beginning of the exponential growth phase, respectively, and t is the time.

Table S5. Materials

Name	# Catalog	Company
Phenol	327125000	Acros Organics
CytoFLEX Daily QC Fluorospheres	B53230	Beckman & Coulter
VersaComp Antibody Capture Bead Kit	B22804	Beckman & Coulter
VersaLyse Lysing Solution	B59266AA	Beckman & Coulter
Sso Advanced Universal SYBR Green Supermix	1725271	Biorad
Albumin CSL 20%	22918180119611	CLS Behring
Privigen Immunoglobulin		CLS Behring
Ultra-Low 24 well	3473	Corning
Eppendorf Tubes® 5.0 ml	0030119380	Eppendorf
85% Glycerol solution	07-3800-07	Hänseler AG
Syringe Filters	FPE-204-030	Jet Biofil
IRDye®800CW Streptavidin	926-32230	Li-Cor
Nucleospin RNA kit	740955.250	Macherey-Nagel
Microtube mesh 40 µm	43-10040-60	PluriSelect life science
GoScript Reverse Trascription System	A5001	Promega
Proteome Profiler Human Adipokine Array Kit	ARY024	R&D Systems
Chloroform	C2432	Sigma-Aldrich
Citric Acid Monohydrate	C1909	Sigma-Aldrich
DAPI	D9542	Sigma-Aldrich
Ethanol	51976	Sigma-Aldrich
Formaldehyde	47608	Sigma-Aldrich
Glutaraldehyde	G6257	Sigma-Aldrich
Guanidine Thiocyanate	50980	Sigma-Aldrich
SDS	74255	Sigma-Aldrich
Sodium Chloride	S7653	Sigma-Aldrich
Triton X-100	X100	Sigma-Aldrich
Urea	U5378	Sigma-Aldrich
PronectinF	Z37866-6	SIGMA-soloHill
SYTO™40 blue fluorescent nucleic acid stain	S11351	Thermo Fisher Scientific
Trypan Blue	15250-061	Thermo Fisher Scientific
TrypLE Select	12563-029	Thermo Fisher Scientific
Glucose Bio (for Cedex Bio)	06 343 732 001	Roche
Lactate Bio (for Cedex Bio)	06 343 759 001	Roche
NH3 Bio (for Cedex Bio)	06 343 775 001	Roche
Via1-Cassette (for NucleoCounter NC200)	941-0012	ChemoMetec
T25 Flask	90026	TPP
T25 Flask	CLS430639	Sigma Aldrich
T75 Flask	CLS430720	Sigma Aldrich
125 mL Disposable Spinner flask	CLS3152	Sigma Aldrich
Collagenase Type B AOF	LS004147	Worthington Biochemical Corp.

Supplementary Materials for the Results Section

Table S6. Percentages of nuclei found in G1, G2, or in S phase.

	**G1**		**S**		**G2**		**APP**	
	BR44	**PNF**	***BR44***	**PNF**	***BR44***	**PNF**	***BR44***	**PNF**
**Day 1**	42.05 ± 3.70 %	37.40 ± 11.57 %	6.10 ± 4.48 %	5.12 ± 3.12 %	12.07 ± 4.03 %	11.43 ± 5.59 %	42.80 ± 17.05 %	42.58 ± 14.39 %
**Day 2**	24.05 ± 4.76 %	31.92 ± 10.07 %	3.16 ± 0.89 %	7.77 ± 2.05 %	8.64 ± 3.05 %	18.04 ± 5.90 %	48.25 ± 6.09 %	82.19 ± 11.96 %
**Day 4**	27.26 ± 8.10 %	41.78 ± 2.07 %	4.60 ± 2.51 %	10.87 ± 3.69	9.90 ±4.15 %	17.30 ± 6.24 %	51.60 ± 8.33 %	67.77 ± 11.27 %
**Day 7**	29.29 ± 9.56 %	42.07 ± 5.95 %	4.15 ± 1.33 %	7.08 ± 0.59 %	9.98 ± 4.41 %	18.34 ± 4.03 %	47.46 ± 5.81 %	61.61 ± 14.83 %

Flow cytometry analysis of CD36 and CD146 expressed by adipogenic-induced hASCs

CD36 is a very useful surface antigen because it labels progenitor cells with a particular susceptibility to undergo terminal adipogenic differentiation. Its expression is correlated with an increase in intracellular neutral lipid content [1,2]. The cell surface marker CD146 is also associated with adipogenic differentiation. Indeed, Graham G. Walmsley et al. showed that hASCs induced to differentiate into adipocytes expressed the CD146 as well as the CD36 markers [3]. Furthermore, Yongting Luo et al. found that macrophagic CD146 promotes foam cell formation and interacts with CD36 to mediate oxidized low-density lipoprotein (oxLDL) uptake [4]. One adipocyte task is to store triglycerides and regulate lipid metabolism. Published studies show that these cells use both CD36 and CD146 for these biological processes. Therefore, both are ideal markers to determine if hASCs spontaneously start to differentiate during the culture time [3,5,6]. Therefore, we wanted to verify if, with our defined xeno- and serum-free culture system, CD36 and CD146 were markers that signal maturation towards the adipogenic line, as described by others. For this purpose, hASCs obtained from three different donors were initially expanded in *UrSuppe* basal medium until confluency was reached. Adipogenic differentiation was then induced with a specifically developed defined medium for ten days. Human ASCs efficiently differentiated into early adipocytes accumulating lipid-containing vesicles that were easily seen under the microscope. After very gently detaching the cells from the cell culture plates, they were labeled with aCD36-APC, aCD146-PE, and analyzed by flow cytometry. The results are shown below in figure S2.

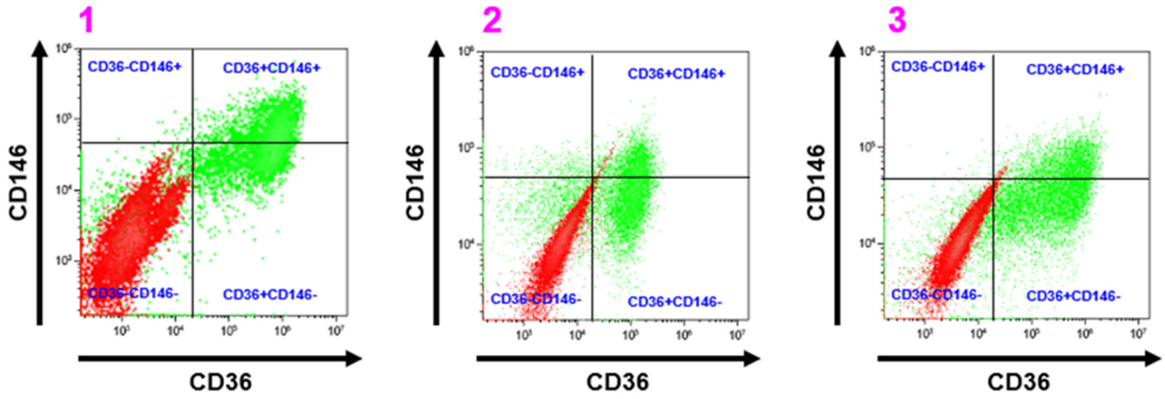

Figure S2. Representative flow cytometry analysis of hASCs induced to differentiate into adipocytes. Early adipocytes from three different donors (plots 1,2, and 3) were stained with aCD36-APC and aCD146-PE. Red populations: Isotype controls. Green populations: Tests with aCD36-APC and aCD146-PE.

We confirm with this test that maturing adipocytes can express both CD36 and CD146 surface antigens, and a significant population is a double-positive for these markers. Flow cytometry with adipose tissue cells is notoriously tricky due to their fragility and high autofluorescence. Indeed, Carolina E. Hagberg *et al.* reported that mature adipocytes' analysis requires a modified flow cytometer specifically adapted for this purpose. This included using a larger nozzle (150 mm diameter), lowering the sheath pressure to 6 psi, and enhancing the detection of large-size events [7]. We worked with a standard flow cytometer, so it is possible that we could not analyze the larger and more mature adipocytes, which probably broke inside the device during the data acquisition. Nonetheless, we confirmed that CD36 and CD146 are valuable markers that signal differentiation/maturation of hASCs into adipocytes.

Flow cytometry analysis of some standard markers expressed by hASCs cultures in 2D or 3D under static conditions

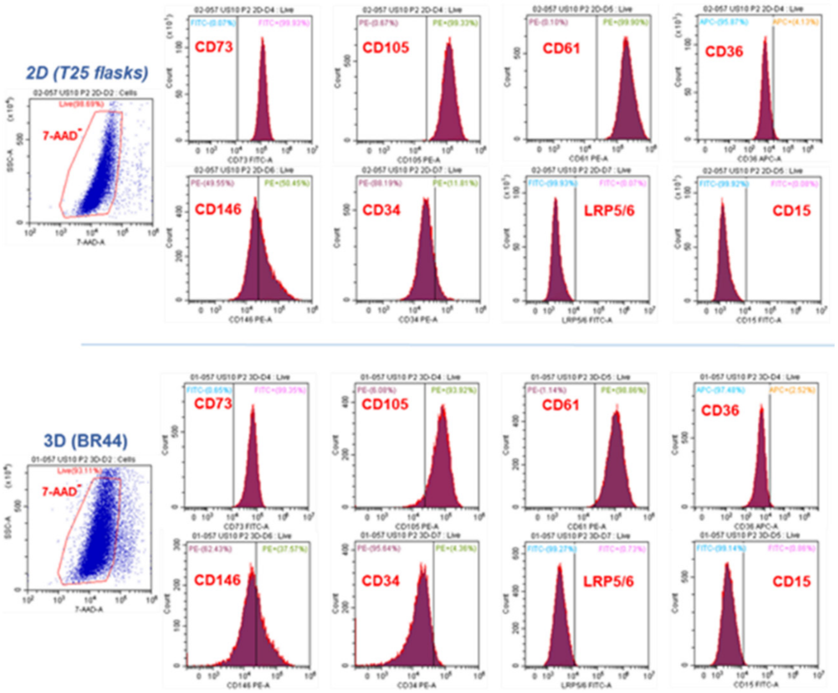

Figure S3. Single-parameter histogram for flow cytometry analysis of expanded hASCs. Cells from donor 057_MaPa at passage P2 stained with labeled antibodies which recognize a panel of standard cell surface markers. Each plot's vertical axis marks the threshold "negative/positive" found with a sample of cells stained with the isotype control antibody. The cells were cultured in SF conditions in standard cell culture vessel (2D, T25 flasks, upper panel) or on the MC prototype *BR44* (3D, lower panel).

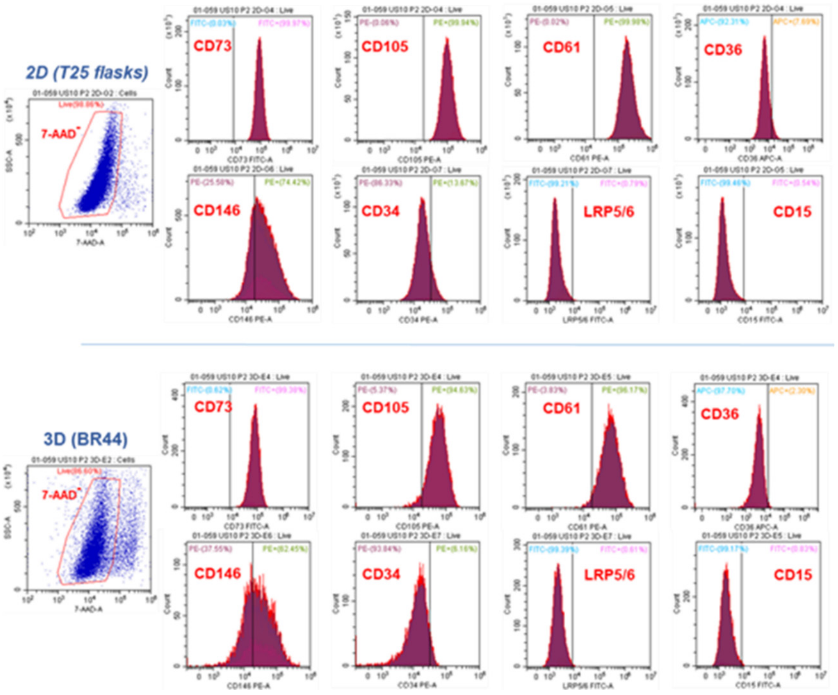

Figure S4. Single-parameter histogram for flow cytometry analysis of expanded hASCs. Cells from donor 059_SeMa at passage P2 stained with labeled antibodies which recognize a panel of standard cell surface markers. Each plot's vertical axis marks the threshold "negative/positive" found with a sample of cells stained with the isotype control antibody. The cells were cultured in SF conditions in standard cell culture vessel (2D, T25 flasks, upper panel) or on the MC prototype *BR44* (3D, lower panel).

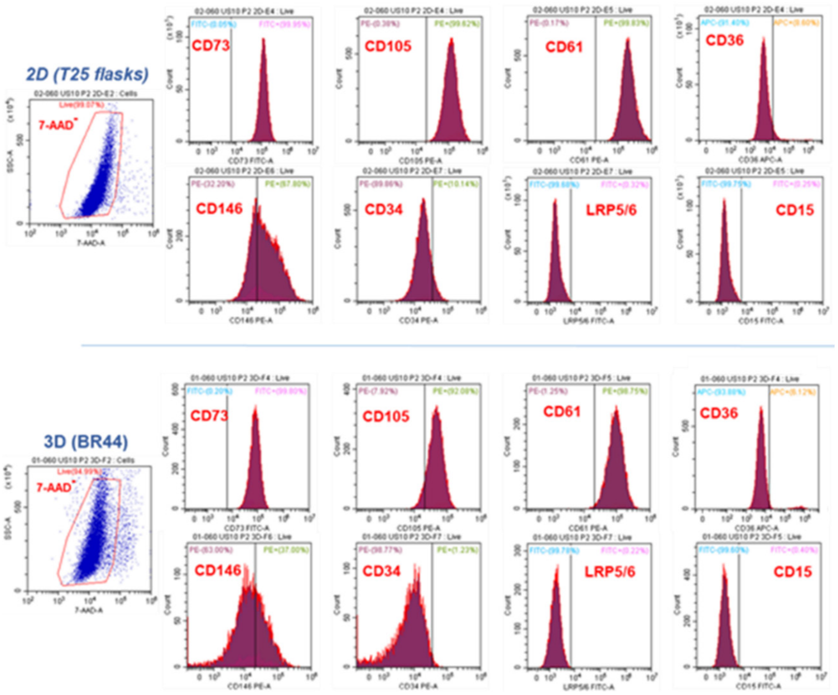

Figure S5. Single-parameter histogram for flow cytometry analysis of expanded hASCs from donor 060_DeMa at passage P2 stained with labeled antibodies recognizing a panel of standard cell surface markers. Each plot's vertical axis marks the threshold "negative/positive" found with a sample of cells stained with the isotype control antibody. The cells were cultured in SF conditions in standard cell culture vessel (2D, T25 flasks, upper panel) or on the MC prototype *BR44* (3D, lower panel).

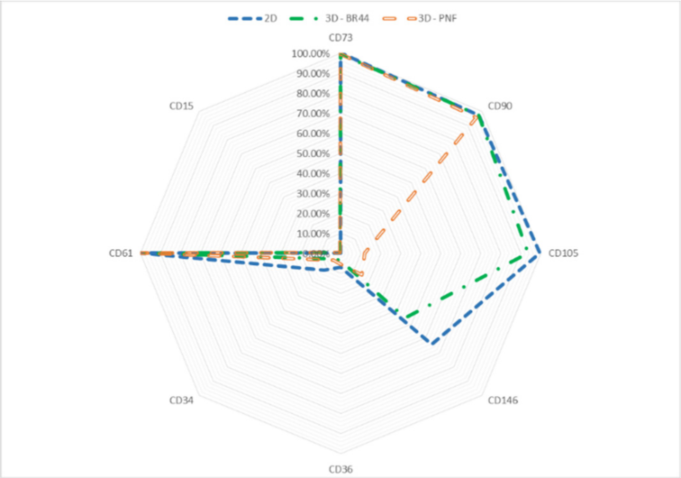

Figure S6. Average flow cytometry expression. "Radar Plot" shows the percentage of positive hASCs for the respectively indicated surface marker for cells grown in 2D (T25 flask, blue line), in 3D on *BR44* (green line), and in 3D on PNF (orange line).

Schematic illustration of adipogenesis

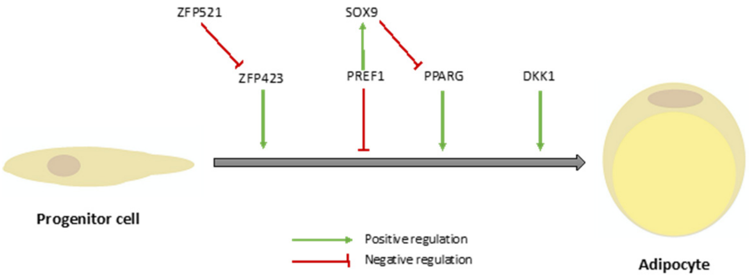

Figure S7. Schematic illustration showing the relationship between some critical factors that positively or negatively controls adipogenesis. Two of them are particularly important: PREF1 is known to inhibit adipogenesis, whereas PPARG is considered the master regulator of adipose tissue development and differentiation. The grey arrow represents the various differentiation stages that lead to a mature adipocyte starting from a progenitor cell. Further information and references about the marker genes used can be found in Table S7, and Table S8.

Table S7. Overview of measured stemness maintenance genes

Name	Description	Reference
PREF1 (DLK1)	Pre-adipocyte factor 1 (Delta-like 1 homolog) is a transmembrane protein that inhibits adipogenesis, and it belongs to the non-canonical Notch ligands family.	Hudak *et al.* [8]<br>Hei *et al.* [9]
SOX9	Sox9 is a member of the HMG-box class DNA-binding proteins and is a Pref1 target.	Wang and Sul [10]
ZFP521	Zinc Finger Protein 521 is a transcription factor, which inhibits adipogenesis.	Chiarella *et al.* [11]<br>Kang *et al.* [12]

Table S8. Overview of measured differentiation regulators/markers

Name	Description	Reference
PPARG	Peroxisome Proliferator-Activated Receptor Gamma is a ligand-dependent transcription factor that is a member of the nuclear hormone receptor superfamily. It plays a crucial role in adipose tissue development and differentiation.	Ahmadian *et al.* [13]<br>Barak *et al.* [14]<br>Rosen *et al.* [15]<br>Tontonoz *et al.* [16]
ZFP423	Zinc Finger Protein 423 is responsible for adipogenic commitment. It induces PPARG expression and terminal adipogenic differentiation.	Gupta *et al.* [17]<br>Gupta *et al.* [18]
DKK1	Dickkopf1 inhibits the Wnt signaling and promotes differentiation.	Christodoulides *et al.* [19]<br>Gustafson and Smith [20]
RUNX2	Runx2 is a transcription factor that is essential for osteoblast differentiation and chondrocyte maturation.	Komori [21]<br>Komori [22]

Table S9. Overview of secreted adipokines and chemokines

Name	Description	Reference
CathepsinD<br>*CathepsinL*	Proteinases: The Cathepsin family plays a role in intracellular protein catabolism and degrade proteins to activate bioactive proteins' precursors in pre-lysosomal compartments.	Kapur and Katz [23]<br>Taleb *et al.* [24]
IGFB-P4<br>*IGFBP-6*<br>*IGFBP-7*	The IGFBP family protein's primary function is regulating the availability of insulin-like growth factors (IGFs) in tissue and modulating IGF binding to its receptors.	Gealekman *et al.* [25]<br>Haywood *et al.* [26]<br>Headey *et al.* [27]
CXCL8/IL8	Interleukin 8 is a pro-inflammatory chemokine/cytokine that induces chemotaxis of granulocytes and stimulates phagocytosis.	Holdsworth and Gan [28]<br>Zlotnik and Yoshie [29]
CCL2/MCP-1	Monocyte Chemoattractant Protein 1 is a small cytokine responsible for recruiting monocytes, memory T cells, and dendritic cells to the inflammation site.	Holdsworth and Gan [28]<br>Zlotnik and Yoshie [29]
M-CSF	Macrophage Colony Stimulating Factor is a secreted cytokine that stimulates macrophage proliferation and activation.	Holdsworth and Gan [28]<br>Zlotnik and Yoshie [29]
MIF	Macrophage Migration Inhibitory Factor is an essential regulator of innate immunity.	Holdsworth and Gan [28]<br>Zlotnik and Yoshie [29]

IL-6	Interleukin 6 acts as a pro-inflammatory cytokine that raises the body's temperature via PGE2 in the hypothalamus and stimulating energy metabolism in fat tissue and muscle.	Holdsworth and Gan [28]<br>Zlotnik and Yoshie [29]
Pentraxin-3/ TSG-14	Proteins of the pentraxin family are involved in acute immunological response.	Kapur and Katz [23]<br>Liu *et al.* [30]
Complement Factor D	Component of the alternative complement pathway of the innate immune system.	Kapur and Katz [23]
Nidogen-1/ Entactin	A structural protein, a component of the basement membrane glycoproteins.	Kapur and Katz [23]
TIMP-1	Metalloproteinase inhibitor. Involved also in promoting cellular proliferation and anti-apoptotic function.	Kapur and Katz [23]
HGF	Growth Factor. Hepatocyte Growth Factor is secreted by mesenchymal stem cells (MSCs) and plays several roles in development, organ regeneration, and wound healing.	Kapur and Katz [23]
VEGF	Growth factor. Vascular Endothelial Growth Factor is a signal protein that stimulates the formation of blood vessels.	Kapur and Katz [23]

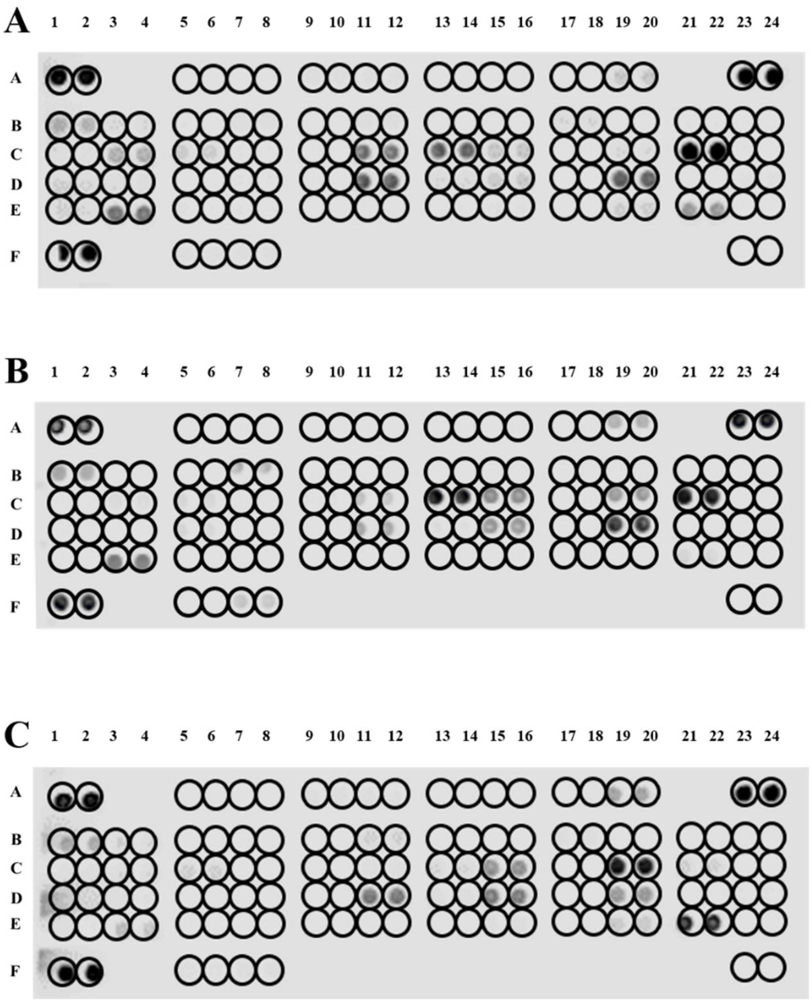

Figure S8. Comparing the secretion profile of hASCs. **A**: Profile of hASCs cultured in the classical static 2D cell culture system. **B**: Profile of hASCs grown on *BR44* MC in static conditions. **C**: Profile of hASCs grown on *BR44* MC in dynamic conditions (spinner flask). Array processing techniques according to the manufacturer of the kit (bio-techne, #ARY024). Evidently, the three secretion patterns are not the same.

Table S10. Human adipokine array

Table/List on the left: Coordinates and explanations of the 58 different spots present on the commercial human adipokine array membrane.

Coordinate	Analyte/Control	Coordinate	Analyte/Control
A1, A2	Reference Spots	C19, C20	IL-6
A5, A6	Adiponectin/Acrp30	C21, C22	CXCL8/IL-8
A7, A8	Angiopoietin-1	C23, C24	IL-10
A9, A10	Angiopoietin-2	D1, D2	IL-11
A11, A12	Angiopoietin-like 2	D3, D4	LAP (TGF-β1)
A13, A14	Angiopoietin-like 3	D5, D6	Leptin
A15, A16	BAFF/BLyS/TNFSF13B	D7, D8	LIF
A17, A18	BMP-4	D9, D10	Lipocalin-2/NGAL
A19, A20	Cathepsin D	D11, D12	CCL2/MCP-1
A23, A24	Reference Spots	D13, D14	M-CSF
B1, B2	Cathepsin L	D15, D16	MIF
B3, B4	Cathepsin S	D17, D18	Myeloperoxidase
B5, B6	Chemerin	D19, D20	Nidogen-1/Entactin
B7, B8	Complement Factor D	D21, D22	Oncostatin M (OSM)
B9, B10	C-Reactive Protein/CRP	D23, D24	Pappalysin-1/PAPP-A
B11, B12	DPPIV/CD26	E1, E2	PBEF/Visfatin
B13, B14	Endocan	E3, E4	Pentraxin-3/SG-14
B15, B16	EN-RAGE	E5, E6	Pref-1/DLK-1/FA1
B17, B18	Fetuin B	E7, E8	Proprotein Convertase 9/PCSK9
B19, B20	FGF basic	E9, E10	RAGE
B21, B22	FGF-19	E11, E12	CCL5/RANTES
B23, B24	Fibrinogen	E13, E14	Resistin
C1, C2	Growth Hormone	E15, E16	Serpin A8/AGT
C3, C4	HGF	E17, E18	Serpin A12
C5, C6	ICAM-I/CD54	E19, E20	Serpin E1/PAI-1
C7, C8	IGFBP-2	E21, E22	TIMP-1
C9, C10	IGFBP-3	E23, E24	TIMP-3
C11, C12	IGFBP-4	F1, F2	Reference Spots
C13, C14	IGFBP-6	F5, F6	TNF-α
C15, C16	IGFBP-rp1/IGFBP-7	F7, F8	VEGF
C17, C18	IL-1β/IL-1F2	F23, F24	Negative Controls

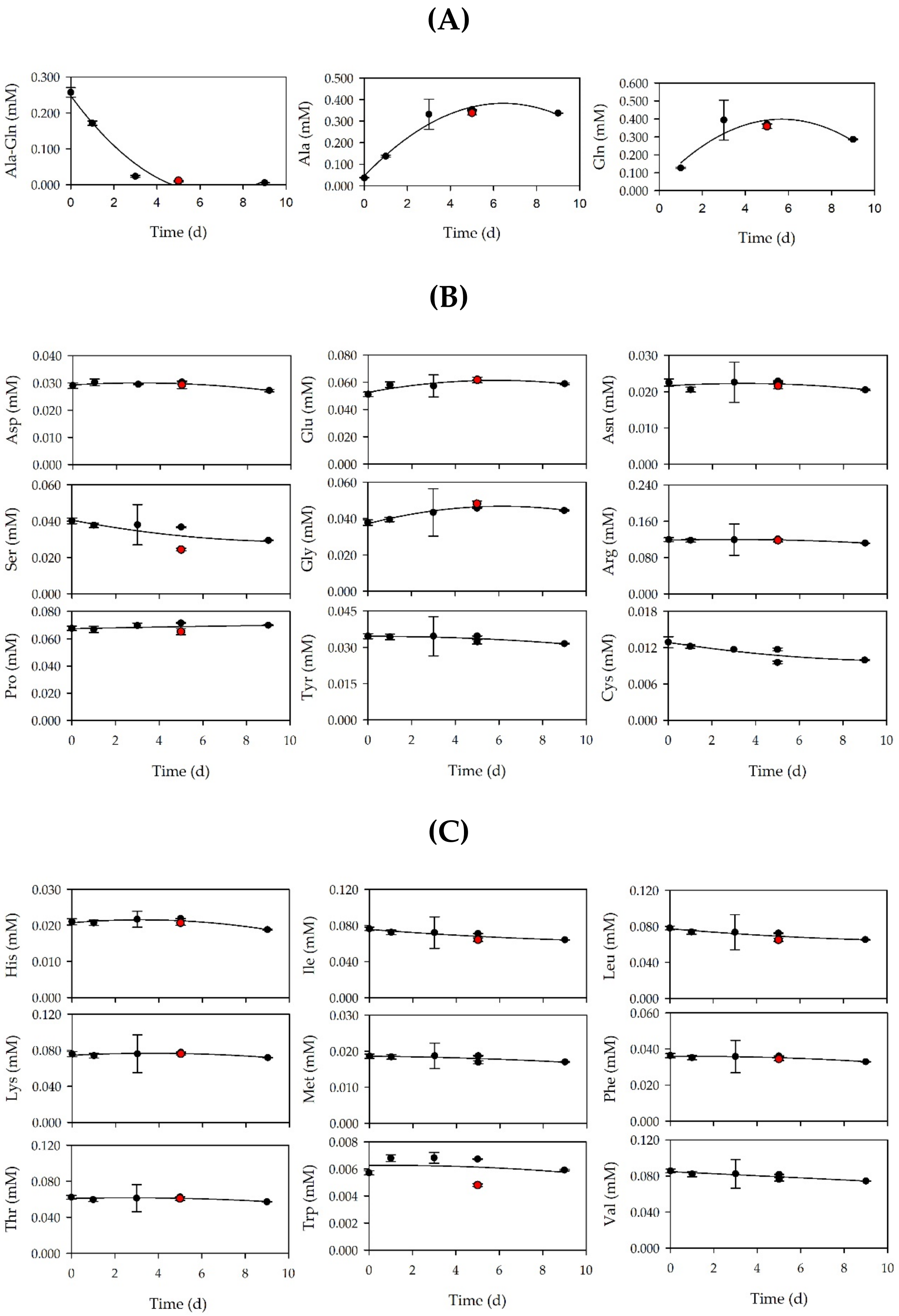

Figure S9. Time-dependent profiles of amino acids consumption: (**A**) Ala-Gln dipeptide. Ala and Gln are generated by slitting the dipeptide. (**B**) Non-essential amino acids. (**C**) Essential amino acids. (●) 3D dynamic cell culture system. (●) Standard planar 2D conditions.

Journal of *Functional Biomaterials*

MDPI

Table S11. Overview of cell culture results achieved in MC-based expansion processes with serum-free cell culture media.

**Cell type**	**Medium**	**MC**	**μ**	**t_d**	**X_{max}**		**EF**	**Cultivation system**	**Ref.**
**[-]**	**[-]**	**[-]**	**[d^{-1}]**	**[h]**	**[10^5 cells/mL]**	**[10^5 cells/cm^2]**	**[-]**	**[-]**	**[-]**
hASC	*UrSuppe* (SFM)	ProNectin (PS)	0.43 ± 0.1	38.7 ± 0.9	±	0.55 ± 0.6	3.1 ± 0.1	125 mL spinner	[31]
ASC52telo (hTERT)	*UrSuppe* (SFM)	ProNectin (PS)	0.56	29.7	7.5	2.08	21.3	125 mL spinner	[32]
ASC52telo (hTERT)	*UrSuppe* (SFM)	*BR44* (PLGA)	0.35	47.5	1.2	n/a	7.0	125 mL spinner	[32]
hASC	*UrSuppe* (SFM)	*BR44* (PLGA)	0.25	67.2	3.2	n/a	8.1	125 mL spinner	[a]
hMSC-TERT	Stem Cell 1 (SFM)	Solohill glass coated	0.31	53.7	2.4	0.45	4.5	100 mL spinner	[33]
hMSC-TERT	Stem Cell 1 (SFM)	Corning EA (PS)	0.65	25.6	2.8	0.52	5.4	100 mL spinner	[33]
hMSC-TERT	Stem Cell 1 (SFM)	Corning Synthemax II (PS)	0.24	69.3	1.8	0.34	3.4	100 mL spinner	[33]
hMSC-TERT	Stem Cell 1 (SFM)	Solohill ProNectin (PS)	0.41	40.6	1.0	0.19	1.9	100 mL spinner	[33]
hBM-MSC	Prime-XV™ (SFM)	Plastic P102-L (PS)	0.42 ± 0.1	39.6 ± 0.9	8.1 ± 0.2	n/a	27.0 ± 0.7	100 mL spinner	[34]
hBM-MSC	Prime-XV™ (SFM)	Plastic P102-L (PS)	0.41 ± 0.1	40.6 ± 0.9	8.5 ± 0.2	n/a	28.3 ± 0.6	ambr 15	[34]
hBM-MSC	Prime-XV™ (SFM)	Plastic P102-L (PS)	0.7	23.8	3.5	n/a	n/a	100 mL spinner	[35]
hBM-MSC	Prime-XV™ (SFM)	Plastic P102-L (PS)	0.52 ± 0.03	31.9 ± 1.7	6.6 ± 0.5	n/a	n/a	250 mL DASGIP DASbox	[36]
hBM-MSC	Prime-XV ™ (SFM)	Plastic P102-L (PS)	0.38 ± 0.02	43.8 ± 2.4	3.0 ± 0.3	n/a	10.0 ± 0.9	100 mL spinner	[37]

[a] = results from the present study, PLGA = Polylactid-co-Glycolid, PS = Polystyrene, EA = Enhanced Attachment

Journal of Functional Biomaterials

MDPI

References

1. Christiaens, V.; Van Hul, M.; Lijnen, H.R.; Scroyen, I. CD36 promotes adipocyte differentiation and adipogenesis. *Biochim. Biophys. Acta - Gen. Subj.* **2012**, *1820*, 949–956, doi:10.1016/j.bbagen.2012.04.001.
2. Gao, H.; Volat, F.; Sandhow, L.; Galitzky, J.; Nguyen, T.; Esteve, D.; Åström, G.; Mejhert, N.; Ledoux, S.; Thalamas, C.; et al. CD36 Is a Marker of Human Adipocyte Progenitors with Pronounced Adipogenic and Triglyceride Accumulation Potential. *Stem Cells* **2017**, *35*, 1799–1814, doi:10.1002/stem.2635.
3. Walmsley, G.G.; Atashroo, D.A.; Maan, Z.N.; Hu, M.S.; Zielins, E.R.; Tsai, J.M.; Duscher, D.; Paik, K.; Tevlin, R.; Marecic, O.; et al. High-Throughput Screening of Surface Marker Expression on Undifferentiated and Differentiated Human Adipose-Derived Stromal Cells. *Tissue Eng. - Part A* **2015**, *21*, 2281–2291, doi:10.1089/ten.tea.2015.0039.
4. Luo, Y.; Duan, H.; Qian, Y.; Feng, L.; Wu, Z.; Wang, F.; Feng, J.; Yang, D.; Qin, Z.; Yan, X. Macrophagic CD146 promotes foam cell formation and retention during atherosclerosis. *Cell Res.* **2017**, *27*, 352–372, doi:10.1038/cr.2017.8.
5. Festy, F.; Hoareau, L.; Bes-Houtmann, S.; Péquin, A.M.; Gonthier, M.P.; Munstun, A.; Hoarau, J.J.; Césari, M.; Roche, R. Surface protein expression between human adipose tissue-derived stromal cells and mature adipocytes. *Histochem. Cell Biol.* **2005**, *124*, 113–121, doi:10.1007/s00418-005-0014-z.
6. Durandt, C.; Van Vollenstee, F.A.; Dessels, C.; Kallmeyer, K.; De Villiers, D.; Murdoch, C.; Potgieter, M.; Pepper, M.S. Novel flow cytometric approach for the detection of adipocyte subpopulations during adipogenesis. *J. Lipid Res.* **2016**, *57*, 729–742, doi:10.1194/jlr.D065664.
7. Hagberg, C.E.; Li, Q.; Kutschke, M.; Bhowmick, D.; Kiss, E.; Shabalina, I.G.; Harms, M.J.; Shilkova, O.; Kozina, V.; Nedergaard, J.; et al. Flow Cytometry of Mouse and Human Adipocytes for the Analysis of Browning and Cellular Heterogeneity. *Cell Rep.* **2018**, *24*, 2746-2756.e5, doi:10.1016/j.celrep.2018.08.006.
8. Hudak, C.S.; Gulyaeva, O.; Wang, Y.; Park, S. min; Lee, L.; Kang, C.; Sul, H.S. Pref-1 marks very early mesenchymal precursors required for adipose tissue development and expansion. *Cell Rep.* **2014**, *8*, 678–687, doi:10.1016/j.celrep.2014.06.060.
9. Hei, S.S. Minireview: Pref-1: Role in adipogenesis and mesenchymal cell fate. *Mol. Endocrinol.* **2009**, *23*, 1717–1725, doi:10.1210/me.2009-0160.
10. Wang, Y.; Sul, H.S. Pref-1 Regulates Mesenchymal Cell Commitment and Differentiation through Sox9. *Cell Metab.* **2009**, *9*, 287–302, doi:10.1016/j.cmet.2009.01.013.
11. Chiarella, E.; Aloisio, A.; Codispoti, B.; Nappo, G.; Scicchitano, S.; Lucchino, V.; Montalcini, Y.; Camarotti, A.; Galasso, O.; Greco, M.; et al. ZNF521 Has an Inhibitory Effect on the Adipogenic Differentiation of Human Adipose-Derived Mesenchymal Stem Cells. *Stem cell Rev. reports* **2018**, *14*, 901–914, doi:10.1007/s12015-018-9830-0.

12. Kang, S.; Akerblad, P.; Kiviranta, R.; Gupta, R.K.; Kajimura, S.; Griffin, M.J.; Min, J.; Baron, R.; Rosen, E.D. Regulation of Early Adipose Commitment by Zfp521. *PLoS Biol.* **2012**, *10,* e1001433, doi:10.1371/journal.pbio.1001433.

13. Ahmadian, M.; Suh, J.M.; Hah, N.; Liddle, C.; Atkins, A.R.; Downes, M.; Evans, R.M. PPAR γ signaling and metabolism : the good , the bad and the future. *Nat. Med.* **2013**, *19*, 557–566, doi:10.1038/nm.3159.

14. Barak, Y.; Nelson, M.C.; Ong, E.S.; Jones, Y.Z.; Ruiz-Lozano, P.; Chien, K.R.; Koder, A.; Evans, R.M. PPARγ is required for placental, cardiac, and adipose tissue development. *Mol. Cell* **1999**, *4*, 585–595, doi:10.1016/S1097-2765(00)80209-9.

15. Rosen, E.D.; Sarraf, P.; Troy, A.E.; Bradwin, G.; Moore, K.; Milstone, D.S.; Spiegelman, B.M.; Mortensen, R.M. PPARγ is required for the differentiation of adipose tissue in vivo and in vitro. *Mol. Cell* **1999**, *4*, 611–617, doi:10.1016/S1097-2765(00)80211-7.

16. Tontonoz, P.; Hu, E.; Spiegelman, B.M. Stimulation of adipogenesis in fibroblasts by PPARγ2, a lipid-activated transcription factor. *Cell* **1994**, *79*, 1147–1156, doi:10.1016/0092-8674(94)90006-X.

17. Gupta, R.K.; Arany, Z.; Seale, P.; Mepani, R.J.; Ye, L.; Conroe, H.M.; Roby, Y.A.; Kulaga, H.; Reed, R.R.; Spiegelman, B.M. Transcriptional control of preadipocyte determination by Zfp423. *Nature* **2010**, *464*, 619–623, doi:10.1038/nature08816.

18. Gupta Rana K. et al. Zfp423 expression identifies committed preadipocytes and localizes to adipose endothelial and perivascular cells. *Cell Metab.* **2012**, *15*, 230–239, doi:10.1016/j.cmet.2012.01.010.

19. Christodoulides, C.; Laudes, M.; Cawthorn, W.P.; Schinner, S.; Soos, M.; O'Rahilly, S.; Sethi, J.K.; Vidal-Puig, A. The Wnt antagonist Dickkopf-1 and its receptors are coordinately regulated during early human adipogenesis. *J. Cell Sci.* **2006**, *119*, 2613–2620, doi:10.1242/jcs.02975.

20. Gustafson, B.; Smith, U. The WNT Inhibitor Dickkopf 1 and Bone Morphogenetic Protein 4 Rescue Adipogenesis in Hypertrophic Obesity in Humans. *Diabetes* **2012**, *61*, 1217–1224, doi:10.2337/db11-1419.

21. Toshihisa, K. Molecular Mechanism of Runx2-Dependent Bone Development. *Mol. Cells* **2020**, *43*, 168–175, doi:10.14348/molcells.2019.0244.

22. Komori, T. Runx2, an inducer of osteoblast and chondrocyte differentiation. *Histochem. Cell Biol.* **2018**, *149*, 313–323, doi:10.1007/s00418-018-1640-6.

23. Kapur, S.K.; Katz, A.J. Review of the adipose derived stem cell secretome. *Biochimie* **2013**, *95*, 2222–2228, doi:10.1016/j.biochi.2013.06.001.

24. Taleb, S.; Cancello, R.; Cle, K.; Lacasa, D. Cathepsin S promotes human preadipocyte differentiation: possible involvement of fibronectin degradation. *Endocrinology* **2006**, *147*, 4950–4959, doi:10.1210/en.2006-0386.

25. Gealekman, O.; Gurav, K.; Chouinard, M.; Straubhaar, J.; Thompson, M.; Malkani, S.; Hartigan, C.; Corvera, S. Control of Adipose Tissue Expandability in Response to High Fat Diet by the Insulin-like

Growth Factor-binding Protein-4. *J. Biol. Chem.* **2014**, *289*, 18327–18338, doi:10.1074/jbc.M113.545798.

26. Haywood, N.J.; Slater, T.A.; Matthews, C.J.; Wheatcroft, S.B. The insulin like growth factor and binding protein family: Novel therapeutic targets in obesity & diabetes. *Mol. Metab.* **2019**, *19*, 86–96, doi:10.1016/j.molmet.2018.10.008.
27. Headey, S.J.; Leeding, K.S.; Norton, R.S.; Bach, L.A. Contributions of the N- and C-terminal domains of IGF binding protein-6 to IGF binding. *J. Mol. Endocrinol.* **2004**, *33*, 377–386, doi:10.1677/jme.1.01547.
28. Holdsworth, S.R.; Gan, P. Cytokines: Names and Numbers You Should Care About. *Clin. J. Am. Soc. Nephrol.* **2015**, *10*, 2243–2254, doi:10.2215/CJN.07590714.
29. Zlotnik, A.; Yoshie, O. The Chemokine Superfamily Revisited. *Immunity* **2012**, *36*, 705–716, doi:10.1016/j.immuni.2012.05.008.
30. Liu, S.; Qu, X.; Liu, F.; Wang, C. Pentraxin 3 as a Prognostic Biomarker in Patients with Systemic Inflammation or Infection. *Mediators Inflamm.* **2014**, *2014*, 1–9, doi:10.1155/2014/421429.
31. Jossen, V.; Muoio, F.; Panella, S.; Harder, Y.; Tallone, T.; Eibl, R. An Approach towards a GMP Compliant In-Vitro Expansion of Human Adipose Stem Cells for Autologous Therapies. *Bioengineering* **2020**, *7*, 77, doi:10.3390/bioengineering7030077.
32. Muoio, F.; Panella, S.; Lindner, M.; Jossen, V.; Harder, Y.; Moccetti, T.; Eibl, R.; Müller, M.; Tallone, T. Development of a Biodegradable Microcarrier for the Cultivation of Human Adipose Stem Cells (hASCs) with a Defined Xeno- and Serum-Free Medium. *Appl. Sci.* **2021**, *11*, 925, doi:10.3390/app11030925.
33. Leber, J.; Barekzai, J.; Blumenstock, M.; Pospisil, B.; Salzig, D.; Czermak, P. Microcarrier choice and bead-to-bead transfer for human mesenchymal stem cells in serum-containing and chemically defined media. *Process Biochem.* **2017**, *59*, 255–265, doi:10.1016/j.procbio.2017.03.017.
34. Rafiq, Q.A.; Hanga, M.P.; Heathman, T.R.J.; Coopman, K.; Nienow, A.W.; Williams, D.J.; Hewitt, C.J. Process development of human multipotent stromal cell microcarrier culture using an automated high-throughput microbioreactor. *Biotechnol. Bioeng.* **2017**, *114*, 2253–2266, doi:10.1002/bit.26359.
35. Rafiq, Q.A.; Ruck, S.; Hanga, M.P.; Heathman, T.R.J.; Coopman, K.; Nienow, A.W.; Williams, D.J.; Hewitt, C.J. Qualitative and quantitative demonstration of bead-to-bead transfer with bone marrow-derived human mesenchymal stem cells on microcarriers: Utilising the phenomenon to improve culture performance. *Biochem. Eng. J.* **2018**, *135*, 11–21, doi:10.1016/j.bej.2017.11.005.
36. Heathman, T.R.J.; Nienow, A.W.; Rafiq, Q.A.; Coopman, K.; Bo Kara; Hewitt, C.J. Development of a process control strategy for the serum-free microcarrier expansion of human mesenchymal stem cells towards cost-effective and commercially viable manufacturing. *Biochem. Eng. J.* **2019**, *141*, 200–209, doi:10.1016/j.bej.2018.10.018.
37. Heathman, T.R.J.J.; Glyn, V.A.M.; Picken, A.; Rafiq, Q.A.; Coopman, K.; Nienow, A.W.; Kara, B.; Hewitt, C.J. Expansion, harvest and cryopreservation of human mesenchymal stem cells in a serum-free microcarrier process. *Biotechnol. Bioeng.* **2015**, *112*, 1696–1707, doi:10.1002/bit.25582.

Publisher's Note: MDPI stays neutral with regard to jurisdictional claims in published maps and institutional affiliations.

© 2020 by the authors. Submitted for possible open access publication under the terms and conditions of the Creative Commons Attribution (CC BY) license (http://creativecommons.org/licenses/by/4.0/).
